# Multi-Omics Approaches Unravel Specific Features of Embryo and Endosperm in Rice Seed Germination

**DOI:** 10.3389/fpls.2022.867263

**Published:** 2022-06-09

**Authors:** Naoto Sano, Imen Lounifi, Gwendal Cueff, Boris Collet, Gilles Clément, Sandrine Balzergue, Stéphanie Huguet, Benoît Valot, Marc Galland, Loïc Rajjou

**Affiliations:** ^1^Université Paris-Saclay, INRAE, AgroParisTech, Institut Jean-Pierre Bourgin (IJPB), Versailles, France; ^2^MBCC Group, Master Builders Construction Chemical, Singapore, Singapore; ^3^Université Paris-Saclay, CNRS, INRAE, Univ Evry, Institute of Plant Sciences Paris-Saclay (IPS2), Orsay, France; ^4^IRHS-UMR1345, Université d'Angers, INRAE, Institut Agro, SFR 4207 QuaSaV, Beaucouzé, France; ^5^Université Paris-Saclay, INRAE, CNRS, AgroParisTech, GQE - Le Moulon, PAPPSO, Plateforme d'Analyse de Proteomique Paris-Sud-Ouest, Gif-sur-Yvette, France; ^6^Chrono-Environnement Research Team UMR/CNRS-6249, Bourgogne-Franche-Comté University, Besançon, France; ^7^Swammerdam Institute for Life Sciences, University of Amsterdam, Amsterdam, Netherlands

**Keywords:** seed, multi-omics, germination, embryo, endosperm

## Abstract

Seed germination and subsequent seedling growth affect the final yield and quality of the crop. Seed germination is defined as a series of processes that begins with water uptake by a quiescent dry seed and ends with the elongation of embryonic axis. Rice is an important cereal crop species, and during seed germination, two tissues function in a different manner; the embryo grows into a seedling as the next generation and the endosperm is responsible for nutritional supply. Toward understanding the integrated roles of each tissue at the transcriptional, translational, and metabolic production levels during germination, an exhaustive “multi-omics” analysis was performed by combining transcriptomics, label-free shotgun proteomics, and metabolomics on rice germinating embryo and endosperm, independently. Time-course analyses of the transcriptome and metabolome in germinating seeds revealed a major turning point in the early phase of germination in both embryo and endosperm, suggesting that dramatic changes begin immediately after water imbibition in the rice germination program at least at the mRNA and metabolite levels. In endosperm, protein profiles mostly showed abundant decreases corresponding to 90% of the differentially accumulated proteins. An ontological classification revealed the shift from the maturation to the germination process where over-represented classes belonged to embryonic development and cellular amino acid biosynthetic processes. In the embryo, 19% of the detected proteins are differentially accumulated during germination. Stress response, carbohydrate, fatty acid metabolism, and transport are the main functional classes representing embryo proteome change. Moreover, proteins specific to the germinated state were detected by both transcriptomic and proteomic approaches and a major change in the network operating during rice germination was uncovered. In particular, concomitant changes of hormonal metabolism-related proteins (GID1L2 and CNX1) implicated in GAs and ABA metabolism, signaling proteins, and protein turnover events emphasized the importance of such biological networks in rice seeds. Using metabolomics, we highlighted the importance of an energetic supply in rice seeds during germination. In both embryo and endosperm, starch degradation, glycolysis, and subsequent pathways related to these cascades, such as the aspartate-family pathway, are activated during germination. A relevant number of accumulated proteins and metabolites, especially in embryos, testifies the pivotal role of energetic supply in the preparation of plant growth. This article summarizes the key genetic pathways in embryo and endosperm during rice seed germination at the transcriptional, translational, and metabolite levels and thereby, emphasizes the value of combined multi-omics approaches to uncover the specific feature of tissues during germination.

## Introduction

Rice is of major importance worldwide, economically, socially, and scientifically. As described for other species, rice germination implies a set of events that begins at the imbibition of the dry mature seed and finishes at the elongation of the embryonic axis (Bewley, 1997). Seed vigor and physiological performance are of paramount importance for proper seedling emergence, increase in crop yield, and reduce the cost of agriculture production (Rajjou et al., 2012; Finch-Savage and Bassel, 2016; Reed et al., 2022). Seed germination is usually defined as a sequential physiological process, including three phases of water uptake (Bradford, 1990). The first phase corresponding to rapid imbibition of dry mature seed, is critical to restore cell activities (*e.g.*, respiration, repair systems, translation) of the seed tissues, namely the embryo and the endosperm. The second phase is a lag period for the fully imbibed seed marked by an intensive metabolic activity with a very characteristic molecular regulation resulting in embryonic cell elongation. Germination is considered complete when the embryo emerges through the testa and cells start to divide in the third phase (Galland et al., 2014). This defined triphasic water uptake profile was also verified for rice seed germination and coleoptile growth (Yang et al., 2007). Upon imbibition, drastic biochemical changes occur within the rice seed tissues. Processes that take place during rice germination are chronologically and differentially regulated depending on imbibition conditions (Magneschi and Perata, 2009; Narsai and Whelan, 2013). For instance, under aerobic conditions, resumption of respiration and of the energy-producing related pathways, such as glycolysis, are activated rapidly upon imbibition (Howell et al., 2006, 2007, 2009). In contrast, under low oxygen conditions fermentative program is initiated to restrict energy consumption and thus produce ATP requisite for cell survival. Indeed, the carbohydrate metabolism can be redirected by transcriptional control into the fermentative branch mainly *via* the accumulation of sucrose synthase, pyruvate decarboxylase, lactate dehydrogenase, and alcohol dehydrogenase (Magneschi and Perata, 2009). Intermediate phases of rice germination include large transcriptional changes that are related to stress responses, energy production, protein degradation, and synthesis, as well as a large number of pathways that sustain elongation of the embryonic axes and the growth of the future seedling (Howell et al., 2009; Narsai et al., 2009). Previous studies highlighted transcriptome, proteome, and metabolome changes in rice during germination; however, these investigations focused mainly on the rice embryo or on the whole grain and the integrated functioning of the endospermic reserve tissue with the diploid embryo for the success of germination and seedling establishment has not yet been taken into consideration. A recent review underlined the coordination between embryo and endosperm and major interactions to ensure rice grain development (An et al., 2020). A multi-omics analysis of dry mature rice compartments emphasized molecular signature related to rice seed quality in a tissue-specific manner (Galland et al., 2017). Only very few studies have been based on detailed imbibition kinetics during the germination process considering both compartments in rice while some experimental evidence have shown in other species that the endosperm is capable to influence the growth of the germinating embryo (Yan et al., 2014). In this study, we deeply investigated the germination process by multi-omics of both the embryo and the endosperm of rice grain (*Oryza sativa* L. var. Nipponbare). We focused on the interpretation of the differential protein accumulation patterns as revealed by label-free shotgun proteomics. Indeed, proteins stored in dry mature seeds and proteins translated during seed imbibition from stored mRNAs have been shown to play a major role in seed germination, both in Arabidopsis (Rajjou et al., 2004) and rice (He et al., 2011; Sano et al., 2012; He and Yang, 2013). A comparison of the proteomic data with corresponding metabolic and transcriptomic data further helped unravel the main functional set of events occurring during rice germination and to have a better understanding of the embryo-endosperm interactions.

## Results

### Rice Germination

**Figure 1:**
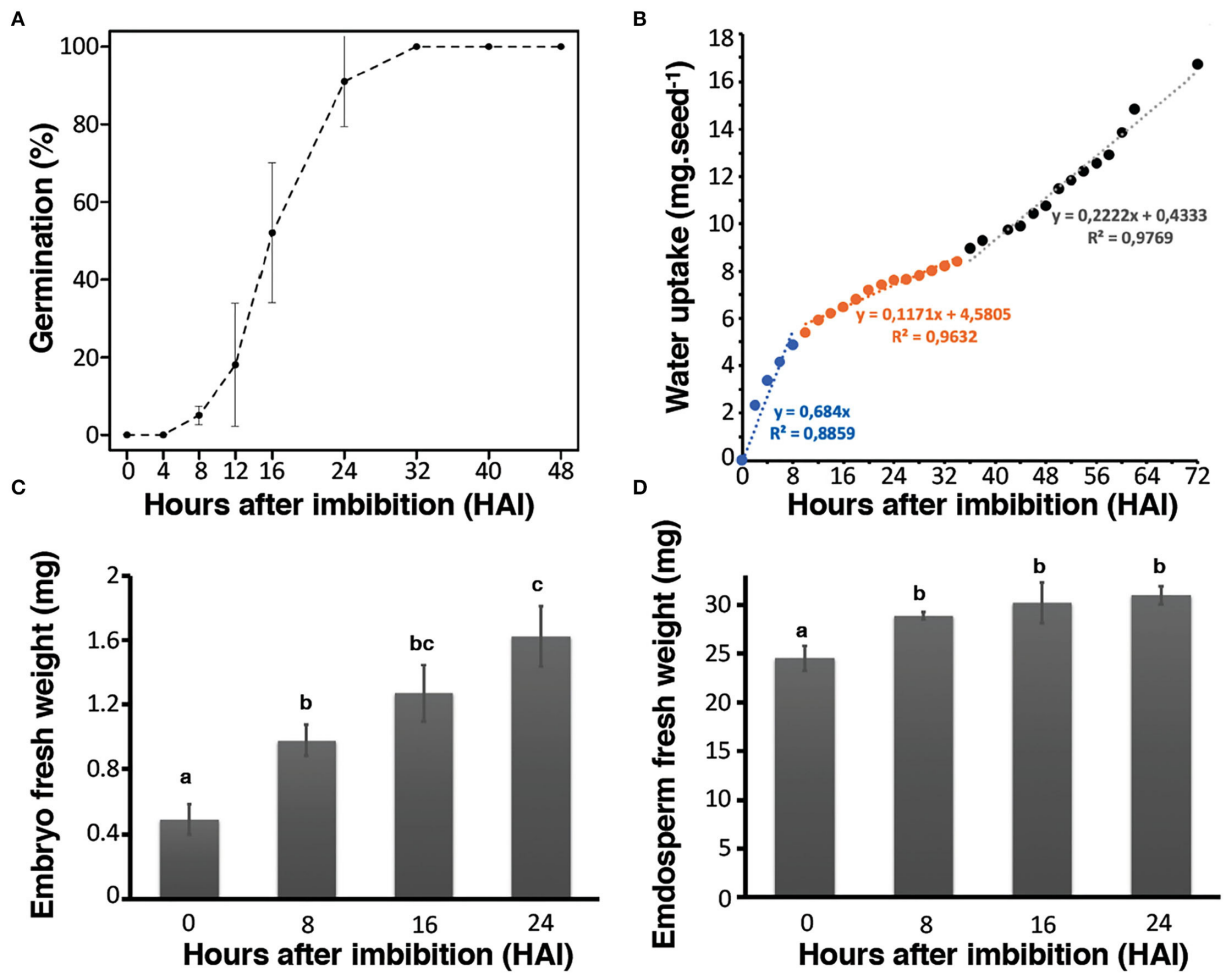
Germination kinetics of Nipponbare rice seeds. Germination assays were carried out at 30°C under dark and continuous oxygenation conditions in triplicate. **(A)** Germination rate of seeds up to 48 h post-imbibition. **(B)** Water uptake in mg of whole rice seeds from 0 to 72 h post-imbibition. The fitted linear models of fresh weight uptake as a function of time are indicated on the graph to highlight the phase I (blue), phase II (orange), and phase III (black) of rice germination. **(C)** Water uptake of isolated embryos; **(D)** water uptake of isolated endosperms. Values are presented as the means ± SD of three replicates. Different letters in **(C)** and **(D)** indicate significant differences (*p* < 0.05, Tukey–Kramer tests).

Rice seed germination kinetics were studied by monitoring coleoptile protrusion that has previously been considered as the observable marker of this physiological process (Howell et al., 2009). Rice seeds used in this study (*Oryza sativa* L. var. Nipponbare) had high vigor and germinative capacity since T_50_ (*i.e.*, time to reach 50% germination) was equal to 16 h after imbibition (HAI) while G_max_ (maximum level of germination) ultimately reached 100% at 32 HAI. In addition, 91% of the seeds germinated within 24 HAI (Figure 1A). Germination can also be followed according to the three phases of water uptake in each seed compartment (*i.e.*, embryo and endosperm) (Bradford, 1990). Accordingly, water uptake measurements were realized from the whole seeds and from both hand-dissected embryo and endosperm during the time course of germination. A very rapid increase in water uptake was observed in the first hours (8–10 HAI) corresponding to phase I of rice germination (Figure 1B). This first stage was followed by a slower period of water uptake corresponding to the phase II (lag period) of rice germination. The transition from phase II to phase III, corresponding to a strong restart of water uptake after germination completion and seedling establishment were visible from beyond 32 HAI (Figure 1B). Interestingly, the patterns of water uptake were remarkably different in the isolated embryo and endosperm (Figures 1C,D). The water content of the embryos steadily increased over the germination time course (Figure 1C), in contrast, the water content in the endosperm rapidly increased up to 8 HAI and then remained stable, consistent with the establishment of the lag period (phase II) (Figure 1D). Previously, water localization was characterized in germinating rice seeds by nuclear magnetic resonance (NMR) (Horigane et al., 2006). The results disclosed first rehydration occurring in the embryo at 2 HAI, which quickly reached the NMR saturation level. Past 8 HAI, the water diffused in the dorsal aleurone layer (transfer cells). Finally, from 8 to 15 HAI, the water slowly diffused into the endosperm. It appears that in the endosperm water distribution evolves convexly toward the center of the seed with a lower signal intensity indicating a lower rate of water penetration (Horigane et al., 2006). The present results are in accordance with these data. Besides physiological approaches, we used combined “multi-omics” (*i.e.*, transcriptomics, proteomics, and metabolomics) approaches thus leading to the most exhaustive tissue-dependent data. Integrating these different levels revealed new and interesting factors impacting rice seed germination (Supplementary Figure S1).

### Germination Phase Transitions in Both Compartments, Embryo and Endosperm, Based on Transcriptomics and Metabolomics

**Figure 2:**
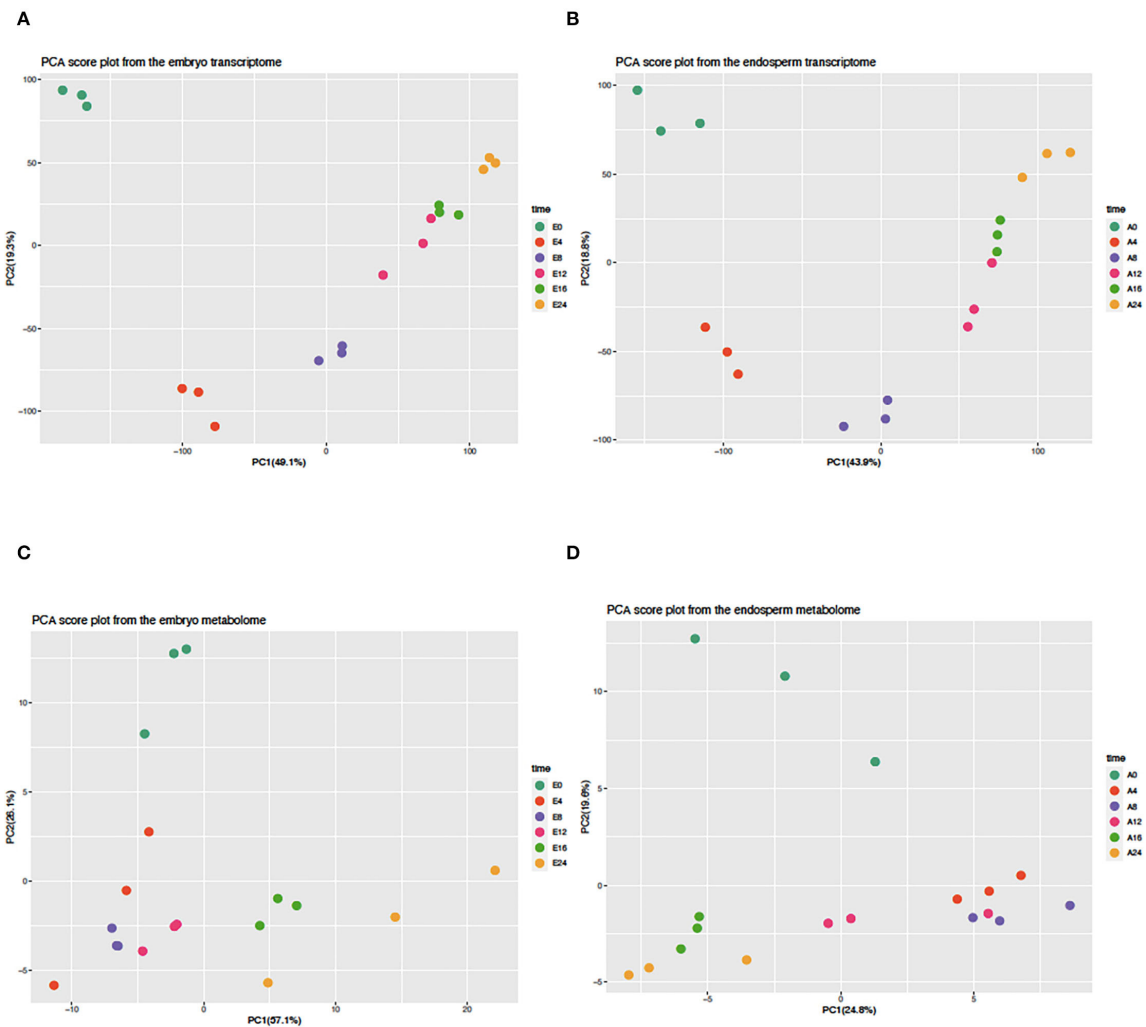
Principal component analysis of embryo and endosperm transcriptomes and metabolomes. The normalized transcript **(A,B)** and metabolite **(C,D)** abundance of isolated embryos **(A,C)** and endosperms **(C,D)** were subjected to a principal component analysis (PCA) of which the first two components are shown in each graph. The percentage of explained variance is indicated along each axis. All values were centered and scaled before computing the PCA.

A transcriptomic approach was performed on both isolated embryos and endosperms during the time course of rice seed germination (0, 4, 8, 12, 16, 24 HAI). This transcriptomic analysis highlighted a significant signal for 22,343 (18,389 loci) and 20,052 locus-specific probes (16,657 loci) in the embryo (E) and endosperm (A), respectively (Supplementary Tables S1A,B). Differentially accumulated transcripts were detected in the embryo (7,665 up and 2,279 down) and endosperm (5,926 up and 2,103 down), respectively, after imbibition (Supplementary Table S2, Supplementary Figure S1). Moreover, during the germination time course, 15,926 rice loci could be detected in both compartments and only 2,463 and 731 transcripts were specifically detected in the embryo or the endosperm, respectively. This detailed transcriptome data was used to plot individual embryo and endosperm samples in a reduced dimension space using a Principal Component Analysis (Figures 2A,B). For both embryo and endosperm, the first component explained nearly half of the total variance (49.1 and 43.9%, respectively) and mainly distinguish early stages (embryo and endosperm at 0 HAI and 4 HAI). On the first component, embryo and endosperm samples at 8 HAI were nearly at the center suggesting that this time point might be the end of the first phase. Therefore, at the mRNA level, it can be thought that the end of phase I could end up as early as 8 HAI in a vigorous rice seed batch in accordance with the findings from the water uptake curve. Surprisingly, on the second component explaining 19.3 and 18.8% of the total variation in the embryo and endosperm transcriptomes (Figures 2A,B), the dry state is closest to the germinated state (E0 and E24, A0 and A24). This points to mRNAs whose levels might be comparable at the beginning and end of the germination process. As previous work did not examine metabolic changes occurring in the endosperm of germinating rice seeds (Galland et al., 2017), we performed a GC-MS based metabolite profiling on both embryo and endosperm during the germination time course. This allowed identifying 380 different metabolites of which 121 were matched to known compounds (Supplementary Tables S3A,C). Besides the fact that the present work is the first description of metabolite profiling in the endosperm of germinating rice seed, these data are currently the most detailed metabolomic study with a detailed time course from dry to germinated seeds. This allowed to highlight 104 and 62 metabolites differentially accumulated during the time course of rice germination in the embryo and the endosperm, respectively (Supplementary Figure S2, Supplementary Tables S3B,D, *p* < 0.05). A PCA analysis on the metabolomic data was also performed for both embryo and endosperm (Figures 2C,D). Contrarily to the transcriptome, here the percentage of variation explained by the first principal component was quite different between the embryo (57.1%) and the endosperm (24.8%). In the embryo, while it seems that PC1 was also related to time after imbibition, the dry state E0 was less well separated from early time points (4, 8, and 12 HAI) while it was easily isolated from later time points (16 and 24 HAI). Since the dry embryo can be distinguished from other time points on the second component (PC2, 26.1% total variation), it suggests that the major metabolic changes take place after 12 h in the embryo. In the endosperm, PC1 explains far less variation (24.8%) than the one in the embryo (57.1%). Yet, it also separates late time points (16 and 24 HAI) from early time points (4, 8, and to a lesser extent 12 HAI). Still, it is also remarkable that the dry endosperm (A0) can be easily separated on the second component PC2 (19.6% of total variation). Overall, in the transcriptome, the first 4 HAI has a dramatic effect on both embryo and endosperm transcriptomes and metabolomes as shown by the clear separation of E0 and A0 samples on PC1 and PC2 (Figure 2). Then we can distinguish a group of early time points (4 and 8 HAI). In the metabolome, the distinction is rather conserved but less sharp suggesting that transcriptome changes are more rapid and consistent than those at the metabolite level.

### Proteome Analyses of Both Compartments, Embryo and Endosperm, in Dry and Germinating Seeds

**Figure 3:**
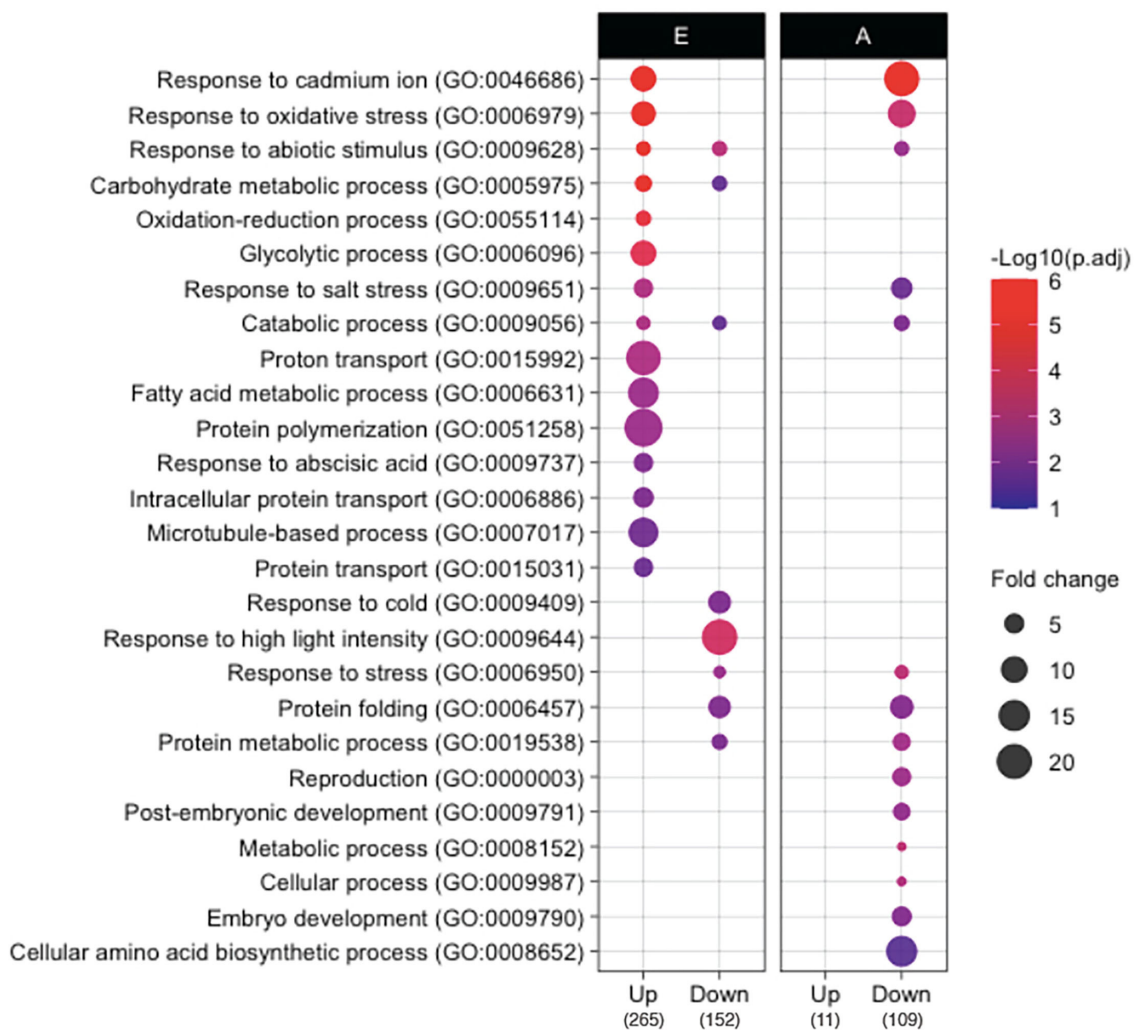
Gene ontology (GO) comparison of proteins that accumulate differently in embryo and endosperm during germination. GO analysis was performed by using the CARMO (http://bioinfo.sibs.ac.cn/carmo/) for differentially accumulated proteins in the embryo (E) and endosperm (A) at 24 HAI compared to 0 HAI (the numbers in bottom brackets of the panel indicate the number of up or down-accumulated proteins). Significant GO terms (adjusted *p*-value < 0.05, Benjamini–Hochberg correction) in “biological process” were obtained from each protein group except for up-accumulated proteins in endosperm since too few proteins (*n* = 11) were up-accumulated in the endosperm. Fold change represents fold enrichment of genes with the given term as compared to ones in the rice genome background.

To elucidate the developmental and metabolic changes accompanying rice seed germination in both isolated embryos and endosperms, a differential proteomic analysis was performed by LC-MS/MS shotgun approach allowing the comparison between dry vs. germinating (24 HAI) seeds (Supplementary Table S4). Thus, 2,419 non-redundant proteins (2,315 in the embryo, 832 in the endosperm, 725 common) with at least two locus-specific peptides in one biological replicate were identified (Supplementary Tables S4A,C). Furthermore, for each protein and in each compartment the protein abundance in dry (0 HAI) and germinating seeds (24 HAI) were compared by calculating the log2 ratio and a *p*-value based on a Student's *t*-test (*p* < 0.05, Supplementary Tables S4B,D). This statistical analysis yielded 417 (19% of the detected embryo proteome) and 120 (16% of the detected endosperm proteome) differentially accumulated proteins during germination in the embryo and the endosperm, respectively. Shotgun proteome analysis revealed 265 up accumulated and 152 down accumulated proteins in embryos during the period from 0 to 24 HAI (Supplementary Tables S5A,C). In the rice endosperm 11 up accumulated proteins and 109 down accumulated proteins were characterized between 0 and 24 HAI (Supplementary Tables S5E,G). To reveal the specific proteome features associated with the germination process, a gene ontology (GO) terms classification of the proteins differentially accumulated in rice embryo and endosperm during germination was also performed (Figure 3). Up accumulated proteins in the embryo during germination belonged mainly to the stress response (GOs:0046686, 0006979, 0009628, 0055114, 0009651, 0009737), carbohydrate metabolism (GOs:0005975, 0006096, 0009056), cytoskeleton (GOs:0051258, 0007017), and transport (GOs: 0015992, 0006886, 0015031) -related classes. These GO categories, characterized on the basis of proteomic analysis of germinating seeds, have been reviewed in detail (He and Yang, 2013; Tan et al., 2013; Czarna et al., 2016). Especially, stress response proteins might accumulate during germination to help the future seedling to cope with environmental conditions as well as the accumulation of proteins involved in carbohydrate metabolism is in accordance with the shift from the quiescent to active metabolic seed state upon imbibition. Down accumulated proteins in the embryo during germination also belonged mainly to the stress response and carbohydrate metabolism but showed specific enriched terms to the detailed categories, such as response to cold (GO:0009404), response to high light intensity (GO:0009644), and protein folding (GO:0006457). It is interesting to note that many of the 152 proteins that were down accumulated in the embryo during germination were previously associated with the maturation program (Zi et al., 2013). For instance, this concerns heat shock proteins (HSPs), LEAs (late embryogenesis abundant) proteins, and proteins related to reactive oxygen species (ROS) homeostasis (Zi et al., 2013). These proteins are definitely involved in desiccation tolerance allowing survival in a dry state. In the rice endosperm, only 11 proteins were up accumulated (Supplementary Table S5E). Functional ontological classification was not possible because of this low number and also because these proteins exhibited very diverse biological functions. However, it is noted that 10 of the 11 up accumulated proteins are specifically identified at 24 HAI as for example for alpha-amylases (AMY1A, LOC_Os02g52710, and AMY3E, LOC_Os08g36900), malate dehydrogenase (LOC_Os08g3372), or defensin DEF8 (LOC_Os03g03810). Ontological classification of the down-accumulated proteins in rice endosperm during germination was again enriched in stress response-related categories (GOs:0046686, 0006979, 0009628, 0009651, 0006950), while specifically over-represented in seed development, such as reproduction (GO:0000003), post-embryonic development (GO:0009791), and embryo development (GO:0009790), as well as cellular amino acid biosynthetic process (GO:0008652). This may highlight the shift from the maturation to the germination process in the endosperm. These GO analyses disclosed that proteins belonging to distinct ontology classes are up or down accumulated in embryos and endosperm during germination, while they also have a common ontology class, such as stress responses. This was also confirmed by mapping the identified proteins on seed-specific function with MapMan (Supplementary Figure S3). Some classes, such as fatty acid synthesis and proton transport, are clearly activated during embryo germination while heat stress-related proteins were down-accumulated in both germinating embryo and endosperm.

### Combined Transcriptome and Proteome Changes in the Embryo During Rice Seed Germination

**Figure 4:**
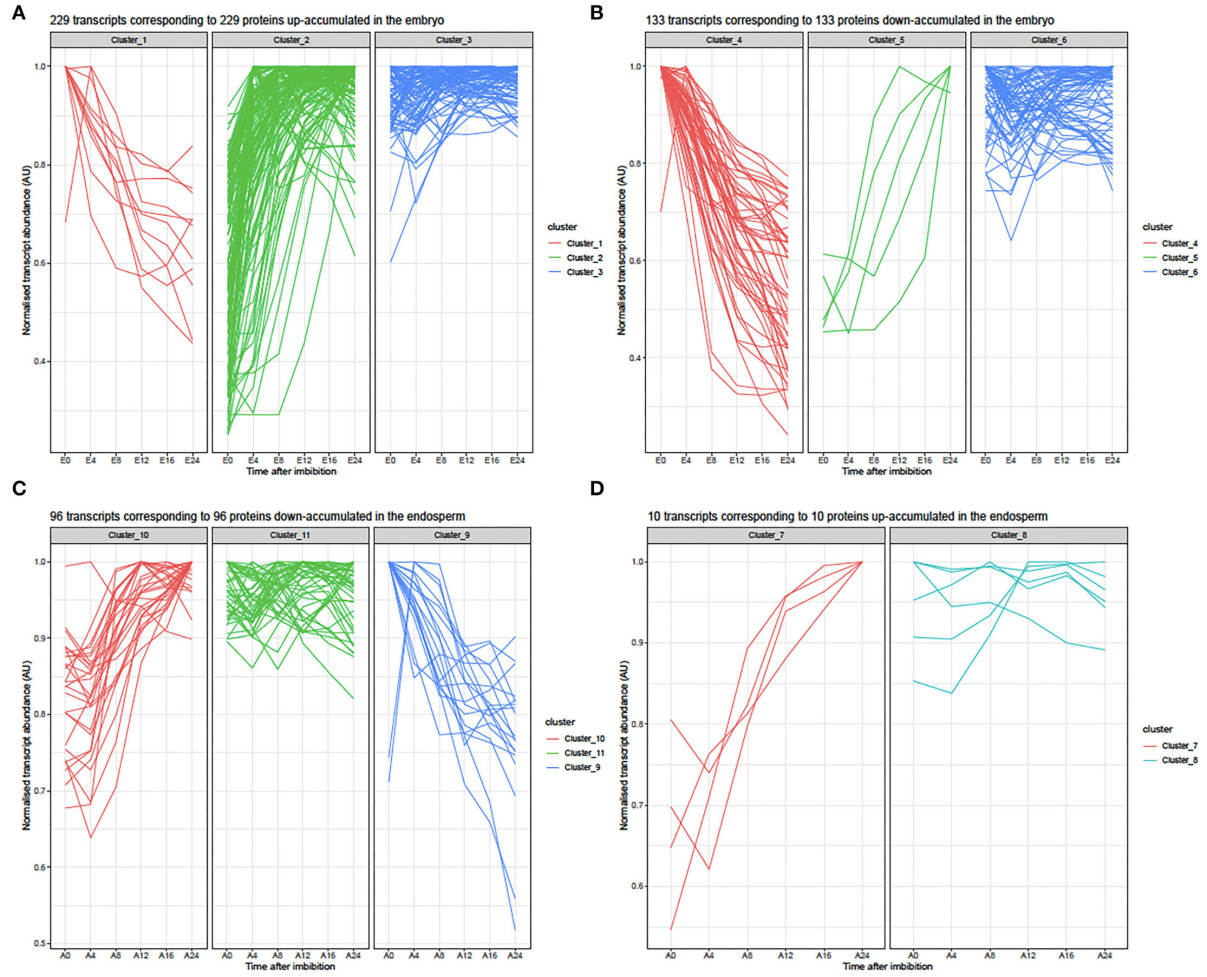
Line plots of transcripts corresponding to embryo or endosperm with significant contrasted accumulations between 0 and 24 HAI. **(A)** Normalized abundances of 229 transcripts detected in the embryo during rice seed germination time course corresponding to 229 proteins up-accumulated at 24 HAI. From the shotgun proteome, 256 up-accumulated proteins were detected, of which only 229 showed changes in the corresponding mRNA abundances. In the embryo transcriptome data, the corresponding mRNA abundance was normalized by their corresponding maximum intensities reached during germination to yield accumulation values comprised between 0 and 1. E, Embryo; E0, dry embryos, E4-E24, dissected embryos from whole rice seed imbibed 4, 8, 12, 16, and 24 HAI. Clusters 1, 2, and 3 are described in Supplementary Table S5B. **(B)** Normalized abundances of 133 transcripts detected in the embryo during the rice seed germination time course corresponding to 133 proteins down-accumulated at 24 HAI. The shotgun proteome evidenced 152 down-accumulated proteins (Supplementary Table S5C). From the embryo transcriptome data, the corresponding mRNA abundances were normalized by their maximum intensities reached during germination to yield accumulation values comprised between 0 and 1. E, Embryo; E0, dry embryos; E4-E24, dissected embryos from whole rice seed imbibed for 4, 8, 12, 16, and 24 HAI. Clusters 4, 5, and 6 are described in Supplementary Table S5D. **(C)** Normalized abundances of 96 transcripts detected in the endosperm during rice seed germination time course corresponding to 96 proteins down-accumulated at 24 HAI. From the shotgun proteome, 109 were down-accumulated proteins at 24 HAI and corresponded to these 91 transcripts (Supplementary Table S5G). From the endosperm transcriptome data, corresponding mRNA abundances were normalized by their corresponding maximum intensities reached during germination to yield accumulation values comprised between 0 and 1. A, Endosperm; A0, dry endosperm; A4-A24, dissected endosperms from whole rice seed imbibed 4, 8, 12, 16, and 24 HAI. Clusters 9, 10, and 11 are described in Supplementary Table S5H. **(D)** Normalized abundances for 10 transcripts detected in the endosperm during rice seed germination time course corresponding to 10 proteins up-accumulated at 24HAI. From the shotgun proteome, 11 were up-accumulated proteins at 24 HAI and corresponded to these 10 transcripts detected in our transcriptome data (Supplementary Table S5F). From the endosperm transcriptome data, the corresponding mRNA abundances were normalized by their corresponding maximum intensity reached during germination to yield accumulation values comprised between 0 and 1. A, Endosperm; A0, dry endosperm; A4-A24, dissected endosperms from whole rice seeds imbibed for 4, 8, 12, 16, and 24 HAI. Clusters 7 and 8 are described in Supplementary Table S5F.

To further unravel the molecular functions involved during rice seed germination, we focused on the interpretation of the differential protein accumulation revealed by label-free shotgun proteomic in relation with the respective transcriptomic data. From the 417 differentially accumulated proteins in embryos during germination, 362 loci (genes with MSU locus IDs) matched with a detected transcript (Supplementary Tables S5B,D). Therefore, for 55 differentially accumulated proteins (36 up- and 19 down-accumulated), no detectable mRNAs were revealed by transcriptomic in both dry and germinating seeds (Supplementary Tables S5B,D). Most of these proteins (47 loci) have no corresponding probes on the chip. Only few of them (8 loci) have no detectable probe in all replicates. Thus, 265 up- and 152 down-accumulated proteins were considered for comparison with their corresponding transcripts (Figure 4; Supplementary Tables S5B,D). From this comparison, three clusters could be defined (Figure 4A). Cluster 1 corresponded to 11 transcripts whose accumulation profiles differed from those observed for the corresponding proteins during rice seed germination (Figure 4A). Thus, these transcripts were abundant in the dry embryos and their accumulation gradually decreased during germination while the corresponding proteins were up-accumulated. Cluster 2 corresponded to 128 transcripts whose accumulation profiles correlated well-with those of the corresponding proteins is an increased abundance over germination time (Figure 4A). Finally, Cluster 3 corresponded to 90 transcripts detected in the dry embryo for which the abundance did not change during germination while the corresponding proteins were up accumulated (Figure 4A). Similarly, from the comparison of down-accumulated proteins with their corresponding transcripts, three clusters were defined (Figure 4B). For the down-regulated transcripts during germination, Cluster 4 corresponded to 54 transcripts whose accumulation profiles correlated well with the observed down-accumulation of the corresponding proteins (Figure 4B, Supplementary Table S5D). Cluster 5 corresponded to 5 transcripts for which accumulation profiles during rice seed germination absolutely differed from the observed accumulation patterns of the corresponding proteins (Supplementary Table S5D). In Cluster 5, these transcripts were progressively accumulated during the time course of germination whereas the corresponding proteins were down accumulated (Supplementary Table S5D). Finally, Cluster 6 corresponded to 74 transcripts detected in the dry embryo and for which the abundance did not change during germination while the corresponding proteins were down-accumulated (Figure 4B, Supplementary Table S5D). Overall, a global comparison of mRNA and protein changes in the embryo and the endosperm shows that both tissues exhibit very poor correlations as previously shown in the dry rice seed (Supplementary Figure S4; Galland et al., 2017).

### Combined Transcriptome and Proteome Changes in the Endosperm During Rice Seed Germination

The present proteome analysis highlighted 121 proteins that were differentially accumulated during germination in the rice seed endosperm (Supplementary Tables S5E,G). It is worth noting that out of them 109 proteins (90%) were down accumulated emphasizing the importance of protein degradation during the germination process. A likely explanation to account for this behavior might be that degradation of proteins accumulated in the mature endosperm is used for fuelling the embryo with free amino acids to boost metabolic restart and seedling establishment upon imbibition. Thus, considering the cereal endosperm as dead tissue, specific proteins may migrate from the aleurone layer to activate physiological processes leading to germination. Interestingly eight down accumulated proteins in the endosperm were up accumulated in the embryo during rice germination (Supplementary Tables S5A,G). All of them belong to the stress response protein family. This is for instance the case for the ATP-synthase (LOC_Os09g08910, LOC_Osg49190) implicated in the respiration pathway, the peptidyl-prolyl cis-trans isomerase (PPI, LOC_Osg01g18120) previously shown to be implicated in protein folding and in the control of the cellular redox state (Laxa et al., 2007; Bissoli et al., 2012), or the glycolytic enzymes, phosphoglucomutase (LOC_Os03g50480) and enolase (LOC_Os10g08550), that may be translocated from the endosperm to the embryo. From the transcriptomic data, 96 transcripts corresponded to the 109 down-accumulated proteins in the endosperm (Supplementary Table S5H). Three clusters could be defined (Supplementary Table S5H and Figure 4C). Cluster 9 corresponded to 20 down accumulated transcripts whose accumulation profiles during germination correlated well with a down-accumulation of their corresponding proteins (Supplementary Table S5H). Cluster 10 corresponded to 29 transcripts whose accumulation profiles completely differed compared to those of the corresponding proteins (Supplementary Table S5H); in cluster 10, these transcripts showed progressive accumulation during the time course of germination whereas the corresponding proteins were down accumulated (Supplementary Table S5H). Finally, Cluster 11 corresponded to 47 transcripts detected in the dry endosperm and for which abundance did not change during germination while corresponding proteins were down accumulated (Supplementary Table S5H). The shotgun proteomic data revealed only 11 proteins that were significantly up accumulated in the endosperm at 24 HAI, the time at which almost all seeds completed germination (Supplementary Table S5E). The corresponding transcripts were grouped according to two major clusters (Supplementary Table S5F, Figure 4D). All these transcripts except for LOC_Os05g49880 were detected in our transcriptome analyses and were strongly accumulated in the rice endosperm at 24 HAI in correlation with the accumulation of corresponding proteins. The main difference between Clusters 7 and 8 is the delay in the time of accumulation. Interestingly, out of the 11 up-accumulated endosperm proteins, 10 of them were germination-specific while the corresponding transcripts were detected both in dry and imbibed endosperm suggesting the occurrence of translational regulation. Altogether, these results emphasized the importance of post-transcriptional and translational controls in rice seed germination.

## Discussion

Seed germination is a complex process that includes, through imbibition, reinitiating cell metabolism from a quiescent to a highly active state, in which a large number of genes are known to be involved. However, there is no integrated analysis of gene expression during rice germination in the two tissues; embryo and endosperm. This study is the first systematic “multi-omics” (transcriptomics, proteomics, and metabolomics) study on both rice embryos and endosperm during germination. Here, we discuss the differences in embryos and endosperm at the multi-omics level, focusing on signaling and hormones, central metabolism, and protein turnover process to provide in-depth insight into the tissue-specific germination process.

### Signaling and Hormones

The proteomic data corresponding to the rice embryo during germination revealed an up-accumulation of the GID1L2-gibberellin receptor (LOC_Os11g13670) and a down-accumulation of CNX1 (LOC_Os04g56620) (Supplementary Table S4), an enzyme involved in the biosynthesis of the molybdenum cofactor (MoCo) essential for ABA biosynthesis (Schwarz and Mendel, 2006), suggesting that as in other seed species these two phytohormones play a central role in rice seed germination. GAs are tetracyclic diterpenoid phytohormones implicated in a wide range of developmental processes, including seed germination (Olszewski et al., 2002; Hedden, 2020). The GID1 GA receptor was previously detected in rice as a soluble receptor implicated in GAs signaling. Mutants affected in the *GID1* gene produce dwarf rice plants (Ueguchi-Tanaka et al., 2005). It is admitted that GID1 action proceeds through active interactions between GID1 and GAs suppressors called DELLA proteins (Ueguchi-Tanaka et al., 2007). Experimental evidence showed that DELLA repression in rice is mainly achieved *via* its degradation as the GID1-GA-DELLA complex is recognized by the 26S proteasome (Ueguchi-Tanaka et al., 2005; Shimada et al., 2008; Sun, 2011). In Arabidopsis, DELLA repression was also shown to be effective through protein-protein interactions between GID1 and DELLA proteins that may be sufficient to block DELLA's repressing activity (Ariizumi et al., 2008). According to Rice Genome Annotation Project, 40 loci are annotated as putative gibberellin receptor GID1. An alignment of the amino acid sequences for these 40 genes, along with that for the three Arabidopsis GID1 orthologs (AtGID1a, b, and c), yields the phylogenetic tree presented in Supplementary Figure S5. All rice GID1 sequences (Supplementary Figure S5) possess a hydrolase domain that corresponds to a putative active GA-binding domain (Shimada et al., 2008). In Arabidopsis, GID1a and GID1c are surmised to be preferentially active during the vegetative development while GID1b is implicated in GA signaling during germination (Ariizumi et al., 2008; Hauvermale et al., 2014). According to amino acid sequence similarities, GID1L2 (LOC_Os11g13670) would be implicated in the GA metabolism during seed during germination. The detection of GID1L2 protein in rice embryos in a germination-specific manner is also supported by the transcriptomic data showing an up-accumulation of the *GID1L2* transcript abundance significantly until 12 HAI in the embryo (Supplementary Table S1A). A down accumulation of the MoCo biosynthesis enzyme CNX1 (LOC_Os04g56620) protein was observed in the imbibed embryo in comparison with the dry state (Supplementary Table S4B) with a decrease in its transcript level upon imbibition (Supplementary Table S1A). This MoCo cofactor is implicated in ABA biosynthesis and thereby its decreased abundance is in accordance with metabolism restart during rice germination as ABA inhibits germination. It is known that the MoCo interacts with the aldehyde oxidase that converts abscisic aldehyde to ABA. Thus, deficient mutants in MoCo biosynthesis do not accumulate ABA (Leydecker et al., 1995; Schwarz and Mendel, 2006). This strongly suggests that the down accumulation of CNX1 protein promotes rice seed germination. Our proteomic analysis also identifies other factors potentially implied in signaling in a germination specific manner, such as the transcription factor HBP-1b (LOC_Os01g06560, Histone Binding Protein-1b). This protein annotated as HBP-1b according to the RGAP (Rice Genome Annotation Project, 7th version) is referred to as OsDOG1L-2 (OsDOG1-like-2), thereby corresponding to an Arabidopsis DOG1 (Delay Of Germination) protein (At5g45830) homolog (Sugimoto et al., 2010). *OsDOG1L-2* gene is a target of the reported preharvest sprouting (PHS) resistance gene, namely Sdr4. In japonica rice cultivar Nipponbare, a highly dormant nearly isogenic line of *Sdr4* (*Seed dormancy 4*) gene containing the indica Kasalath allele (NIL[*Sdr4-k*]) over-express *OsDOGL1-1* but the *OsDOGL1-2* expression remained unaffected. *Sdr4*, is considered as a major regulator involved in seed dormancy and domestication of rice (Sugimoto et al., 2010). From our present omics data, HBP-1b/OsDOGL1-2 transcript and protein abundance increase significantly in the embryo during germination. Interestingly, HBP-1b was located inside of a QTL region for seed dormancy (Li et al., 2011) and or salinity tolerance (Lakra et al., 2015). The novel finding observed here showing OsHBP-1b accumulation in the embryo during germination might reveal a novel function, distinct from DOG1's, of this TF belonging to the bZIP family in rice germination. Recent work showed that transgenic rice over-expressing *OsHBP-1b* exhibits better germination capacity, higher shoot growth, and higher fresh weight under salinity stress than the wild type (Das et al., 2019). Further studies are needed to establish the function of DOG1-like genes in the modulation of rice seed germination and vigor.

The endothelial differentiation-related factor 1 (EDF1, LOC_Os06g39240) belonging to the MBF1 (multiprotein bridging factor 1) transcription factor family was also identified specifically in imbibed rice embryo, at the time of germination completion (24 HAI). OsEDF1 is orthologous to AtMBF1c (At3g24500) with 70% of amino acid sequence homology. MBF1 is known as an evolutionary conserved transcriptional co-activator that mediates transcriptional activation by bridging between an activator and a TATA-box binding protein (Takemaru et al., 1997; Jaimes-Miranda and Chávez Montes, 2020). In Arabidopsis, *mbf1c* mutants were more sensitive to heat stress than are wild-type plants (Suzuki et al., 2008). In contrast, transgenic rice over-expressing wheat *MBF1c* was more tolerant to heat stress (Suzuki et al., 2005; Qin et al., 2015). In seeds, MBF1c is suggested to act as a positive regulator of ABA degradation during the early steps of germination (Di Mauro et al., 2012). To our knowledge, no data describing MBF1 protein action in rice seeds have been reported yet. We found that the EDF1/MBF1c protein was specifically accumulated in germinating embryos, although the transcriptomic data indicated a decreased level of the corresponding transcript Supplementary Tables S1A, S4A). This result suggested that the functional activation driven by *EDF1/MBF1* is under translational control during rice germination. The ABA content in rice seeds decreases rapidly after few hours of imbibition (Song et al., 2020) concomitantly with an increased expression of the ABA catabolism genes (*OsABA8ox2, OsABA8ox3*) (Zhu et al., 2009; Wang et al., 2021), supporting the finding that as in Arabidopsis, rice MBF1 acts as a positive regulator of ABA degradation (Di Mauro et al., 2012), thereby favoring germination. Interestingly, this gene is also described as an ethylene-response transcriptional co-activator (ERTCA) and could play a central role in the crosstalk between ABA and ethylene to control germination vigor and stress responses (Arc et al., 2013; Jaimes-Miranda and Chávez Montes, 2020). In Arabidopsis, ethylene boosts protein and mRNA levels of plant defensins to cope with pathogens (Penninckx et al., 1998). In the endosperm, the defensin DEF8 was up accumulated at 24 HAI. The OsDEF8 previously displayed antimicrobial activity against phytopathogens, such as *Xanthomonas oryzae* and *Fusarium oxysporum*, indicating a role in disease control (Tantong et al., 2016; Weerawanich et al., 2018). The specific accumulation of DEF8 in rice endosperm during germination suggests a protecting role of the embryo by this compartment through antimicrobial peptide production.

### Central Metabolism

#### Tricarboxylic (TCA) and Glyoxylate Cycles for Energy Feeding

**Figure 5:**
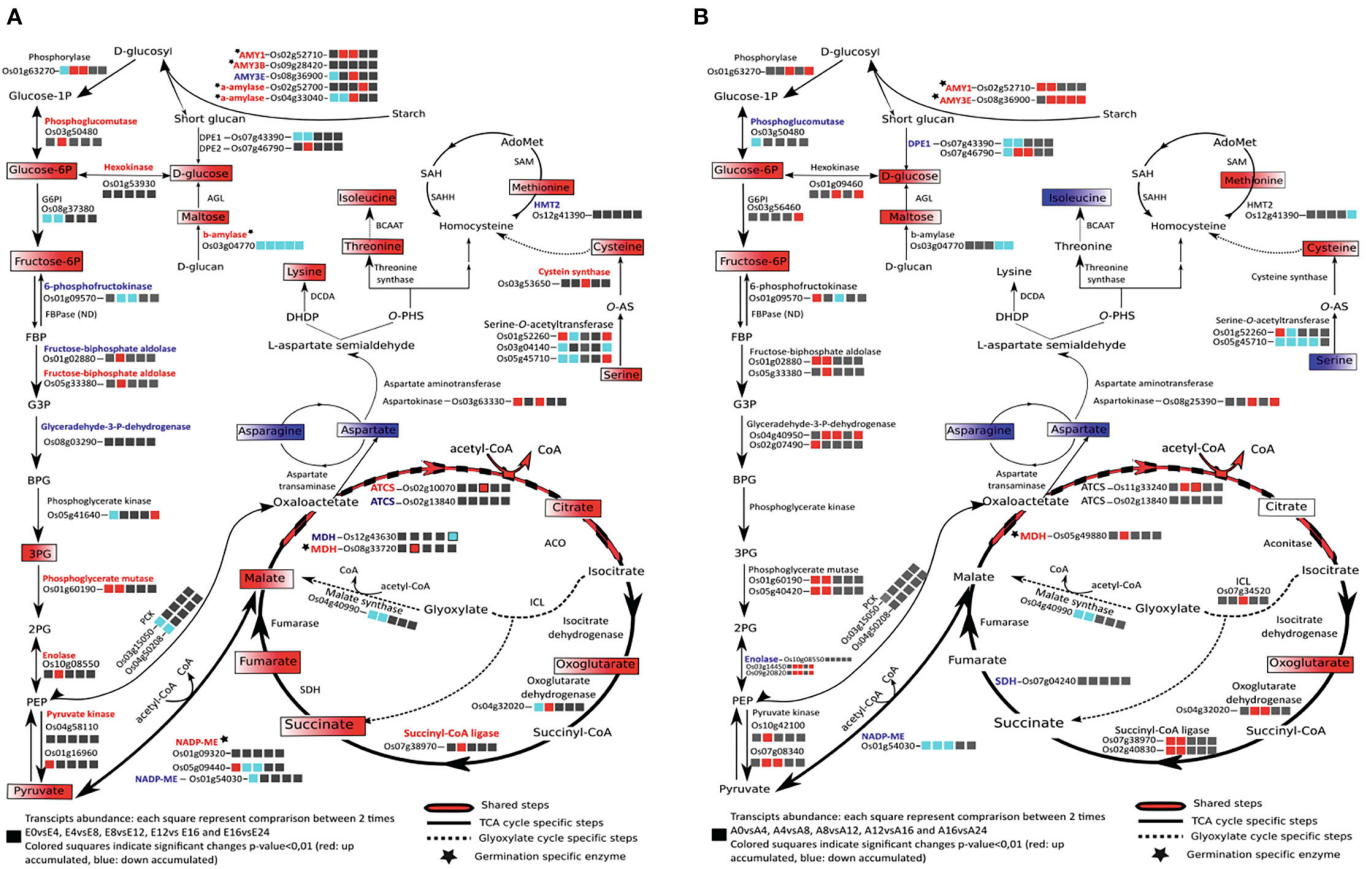
Metabolic pathways related to seed glycolysis/neoglucogenesis, energy, and amino acid metabolisms in rice embryo **(A)** and rice endosperm **(B)** comparing mature dry and germinated seeds. Up and down-accumulated metabolites during germination are displayed, respectively, in red and blue boxes. Proteins present in the total embryo proteome are indicated in black if not detected or if their abundance change was not significant. ACO, aconitase; AGL, alpha-glucosidase; ATCS, citrate synthase; BCAAT, branched-chain-amino-acid aminotransferase; DCDA, diaminopimelate decarboxylase; DHDP, dihydrodipicolinate; DPE, depolymerizing enzyme; FBP, fructose-1,6-bisphosphate; G3P, glycerate-3-phosphate; HMT2, homocysteine *S*-methyltransferase; ICL, isocitrate lyase; MDH, malate dehydrogenase; NADP-ME, NADP-malic enzyme; *O-*PHS, *O-*phospho-L-homoserine; PCK, phosphoenolpyruvate carboxykinase; PEP, phosphoenolpyruvate; 3PG, 3-phosphosphoglycerate; *O-*AS-, *O-*acetylserine; SAH, *S-*adenosyl-L-homocysteine; SAHH, *S-*adenosyl-L-homocysteine hydrolase; SAM2, *S-*adenosylmethionine synthetase; SDH, succinate dehydrogenase; TCA, tricarboxylic acid.

During rice seed germination, respiration starts just after 1 HAI and mitochondrial differentiation starts after 8 HAI (Howell et al., 2006). Plastidic and cytosolic glycolysis together with the mitochondrial TCA cycle are the main components of the respiratory metabolism that supply carbon and energy to cells (Fernie et al., 2004). The accumulation of AMY1A and AMY3E in rice endosperm during germination indicates the catalysis of starch breakdown providing substrate for the glycolysis and the pentose phosphate pathway and promoting energy production and reducing power for embryo germination. The TCA and glyoxylate cycle intermediates during rice germination were identified by the present metabolic analysis and all the identified metabolites were highly accumulated in the germinating embryos (Supplementary Table S3A, Figure 5A) in concordance with a previous study (Howell et al., 2009). In endosperm, 61 metabolites were identified as being significantly and differentially accumulated (Supplementary Table S3C). The highlighted metabolic pathways indicate an up accumulation of the glycolysis by-products in endosperms rapidly after imbibition; this may be correlated to starch degradation (major compound) (Figure 5B). Identification of the associated enzymes differentially accumulated between embryo and endosperm may pinpoint key steps involved in the activation and maintenance of the energetic flux for germination. NADP-malic enzymes (LOC_Os01g09320 and LOC_Os05g09440), citrate synthases (ACTS, LOC_Os11g47330, LOC_Os02g13840), and malate dehydrogenase (MDH, LOC_Os08g33720) specific isoforms were more accumulated in the germinated embryos than in the endosperm tissue. Interestingly, two plastidic isoforms of the NADP-malic enzyme (LOC_Os01g09320 and LOC_Os05g09440) were present specifically at the germinated state (Supplementary Table S5A; Figure 5A). In contrast, the abundance of the NADP-malic enzyme encoded by the *LOC_Os01g54030* gene significantly decreased at 24 HAI in both embryo and endosperm. NADP-malic enzymes catalyze the reversible and oxidative decarboxylation of malate to pyruvate. In the Arabidopsis genome, four genes encode the NADP-ME, namely three cytosolic NADP-ME 1,2,3 and a plastidic NADP-ME4, while according to the rice genome, seven isoforms are annotated. The Arabidopsis NADP-ME2 (AT5G11670) presents a protein sequence similarity of about 81% with the rice NADP-ME (LOC_Os05g09440). Transcript abundance of the NADP-malic enzyme isoform LOC_Os05g09440 was significantly up accumulated at the early stages of imbibition (0-4 HAI) (Figure 5A; Supplementary Table S1A), which may be correlated to the specific role of this enzyme in oxidative stress response, as previous studies reported an up accumulation of the *NADP-ME2* (AT5G11670) in Arabidopsis leaves submitted to oxidative stress, although accumulation of the enzyme was shown to be a non-limiting factor of the stress response (Li et al., 2013). Oxidative burst occurring during imbibition may be correlated to the increase of this *NADP-*ME transcript in the earliest steps of germination (Liu et al., 2007). The activation of the TCA cycle may explain the high accumulation of the NADP-ME that catalyzes the oxidative decarboxylation of malate to produce pyruvate and then feed the TCA cycle (Chang and Tong, 2003; Chen et al., 2019). The same behavior was observed for the malate dehydrogenase (MDH) where two isoforms (LOC_Os12g43630 and LOC_Os08g33720) were identified as being significantly up (LOC_Os08g33720) and down (LOC_Os12g43630) accumulated in the embryo (Supplementary Tables S5A,C; Figure 5A). Finally, it is admitted that the cyclic flux is maintained to ensure the respiratory metabolism *via* the acetyl CoA input where the oxaloacetate and acetyl CoA condensation is the starting reaction activating the TCA cycle flux (Sweetlove et al., 2010). However, a TCA cycle connection with the glyoxylate cycle and the aspartate family super pathway is not necessarily maintained as a circular flux especially under stress conditions to sustain adequate levels of ATP (Sweetlove et al., 2010). This observation may pinpoint a metabolic orientation in germinating seeds depending on seed storage compound composition and actual metabolic requirements. In Arabidopsis seeds, glyoxylate and TCA cycle intermediates and enzymes are up accumulated during germination (Fait et al., 2006; Galland et al., 2014). In germinating rice seeds, as in Arabidopsis, fructose-6P, glucose 6P, and almost all the TCA and glyoxylate cycle intermediates presently identified are accumulated. The TCA and glyoxylate cycles exhibit a diverse set of features rendering this machinery much more able to deal with substrates supply (van Dongen et al., 2011).

#### Amino Acid Metabolism

In addition, to provide the monomeric precursors of proteins, amino acids are the precursor form of carbon and nitrogen and play a key role in the energy supply. Indeed, in non-photosynthetic organisms, amino acid catabolisms play a role to feed the tricarboxylic cycle (TCA) as an alternative way to maintain energy supply (Galili, 2011). Amino acid metabolism also plays as an important role during stress response where it is admitted that transcriptomic changes include the repression of amino acid biosynthesis genes and stimulation of genes implicated in the catabolism of some amino acids (Galili, 2011; Wang et al., 2018). From our GC-MS metabolomic data, 29 and 15 amino acids were detected in the rice embryo and endosperm, respectively, as being significantly and differentially accumulated during germination. In rice embryo, most of the amino acids were drastically accumulated at 24 HAI suggesting a burst of energy mobilization for post-germination phases and plant development (Supplementary Tables S3A,C, Supplementary Figure S6). In the endosperm, eight amino acids were down accumulated and seven were up accumulated during rice germination; however, fold changes are quite low varying from 0.3 to 1.7 (Supplementary Table S3C). This may indicate that most amino acid changes observed here are those of the aleurone layer, which contains more proteins than the starchy endosperm. Interestingly, aspartate abundance decreased very rapidly upon imbibition in both embryo and endosperm, suggesting consumption of this amino acid pool to feed the aspartate pathway (Figure 5, Supplementary Figure S6). This observation is one of the main differences between the two plant models, Arabidopsis and rice, since in Arabidopsis the aspartate level increases during seed germination (Fait et al., 2006). The aspartate-degradation super pathway leads to the formation of methionine, lysine, and threonine. Each of these amino acids plays a key role in the cell metabolism and the organism surviving. From the present “omic” data, the aspartate pathway can be redrawn to show transcripts, proteins, and metabolites abundance in the embryo during germination (Supplementary Figure S7). As only few changes were observed in the endosperm, in the following, we will only focus on the germinating embryo aspartate pathway. Aspartate-derived amino acids were up accumulated in germinated embryos with ratios varying from 2 for the threonine to 5 for the isoleucine (Supplementary Figures S6, S7). It is noted that while lysine, threonine, and methionine drastically increase (Figure 5A; Supplementary Table S3A, Supplementary Figure S6), most enzymes of this pathway were not differentially accumulated during germination (Figure 5 and Supplementary Figure S7; Supplementary Table S4B). Only three enzymes displayed significant changes at 24 HAI (Supplementary Figure S7; Supplementary Table S4B). Aspartate dehydrogenase (LOC_Os03g55280) and aminotransferase (LOC_Os10g25130) were significantly up accumulated during germination in accordance with the apparent activation of this pathway. Homocysteine-*S*-methyltransferase (methionine synthase) was significantly down accumulated (2-fold change) despite the increase in methionine level suggesting a regulated circular flux of synthesis/catabolism.

The sulfur amino acid metabolism is a major hub to the physiology of seed germination, allowing connecting housekeeping metabolic activity and hormonal regulation (Rajjou et al., 2012). Methionine is the initiator of protein elongation and is the precursor of *S*-adenosyl-methionine (SAM) the activated form of methionine and the universal methyl donor for a myriad of trans methylation reactions (Ravanel et al., 1998; Rahikainen et al., 2018). Methionine and SAM are involved in different central reaction pathways. Indeed, methionine biosynthesis is directly linked to the folate, cobalamin, and pyridoxine pathways and methionine-derived SAM is the substrate in most preponderant trans methylation reactions, including protein repair, as well as in DNA/RNA and histone methylation, Gas, ethylene, and biotin biosynthesis pathways rendering methionine a key regulator metabolite (Amir, 2010; Sauter et al., 2013; Huang et al., 2019). Methionine-derived ethylene is also a crucial hormone being involved in many pathways as plant growth and immune responses (Broekgaarden et al., 2015; Dubois et al., 2018). Methionine is required for protein synthesis in seed germination and seedling establishment both in Arabidopsis (Rajjou et al., 2004) and rice (Sano et al., 2012). It is noted that although chemical inhibition of methionine synthesis substantially delays seed germination in Arabidopsis but does not completely block this process while exogenous L-Met promotes germination (Gallardo et al., 2002; Ju et al., 2020). It is therefore possible that methionine accumulation through proteome renewal (*i.e.*, catabolism of proteins stored in the dry mature seeds) contributes significantly to this methionine requirement of rice embryo and therefore *de novo* methionine may not be the preferred mechanism, at least in the early phases of seed germination.

### Protein Turnover

#### Protein Synthesis

During germination, the balance between synthesis and degradation of proteins is a highly regulated turnover process rendering possible the biological and metabolic switch from quiescence to active metabolism upon imbibition. The cellular proteome is established by the principal translation machinery; Cytosolic ribosomes consist of large 60S and small 40S subunits. During translation, recruitment of the 40S subunit starts at the 5′ end of mRNAs, which are bound to eukaryotic translation initiation factors, namely, a cap binding protein (eIF4E) and a helicase (eIF4A). A circle bridge is then formed between the 3′ and 5′ ends of the mRNA thus stimulating the translation process through the association of the eIF4G and eIF4B (Browning and Bailey-Serres, 2015). The eIF4B enhances the helicase activity of eIF4A, thereby determining the unwinding of 5′ mRNA cap's secondary structure (Bi et al., 2000). A total of 30 translation factors, including two eIF4B isoforms (LOC_Os02g24330 and LOC_Os02g38220), were identified by our proteomics, and LOC_Os02g24330 was up-accumulated at 24 HAI in rice embryos. These suggest that the translation activity of germinating rice embryos may be mainly managed through eIF4B (LOC_Os02g24330). Ribosomal proteins are the elementary components of ribosomes together with rRNA (Weis et al., 2015). Regulation of eukaryotic translation is also carried out by the interaction of the translation initiation factors with ribosomal proteins (Muench et al., 2012). In the present work, 13 ribosomal proteins were significantly up accumulated in rice embryos in which two (LOC_Os03g26860, LOC_Os05g48050) were strictly detected in the germinated embryos (24HAI) (Supplementary Table S4A). Interactions between ribosome and translation initiation factors orientate spatial translation and subsequent protein synthesis. Protein synthesis was detected in all rice compartments suggesting that translation could occur even in the isolated starchy endosperm (Galland et al., 2017). In agreement with this, the shotgun analysis data revealed an accumulation of two ribosomal proteins (40S ribosomal protein (LOC_Os02g48660) and a 60S ribosomal protein (LOC_Os02g48660) specifically in the endosperm at 24 HAI, although the most active compartment for protein synthesis is the embryo (Yang et al., 2007; Kim et al., 2009; He and Yang, 2013; Galland et al., 2017). Endosperms with conserved aleurone layer exhibited a slightly higher labeling signal than the starchy endosperm, suggesting an active protein synthesis from the aleurone layers during rice seed germination. Inhibition of translation is deleterious for rice germination while *de novo* transcription is not required (Sano et al., 2012, 2019). This suggests, as in Arabidopsis (Rajjou et al., 2004), a requirement for protein synthesis from stored mRNAs accumulated during seed development. The molecular mechanism of seed germination using stored mRNAs has been reviewed in detail recently (Sajeev et al., 2019; Sano et al., 2020). Particularly, the selective translation mechanisms of stored mRNAs could be one of the reasons for those levels of transcript changes do not always correlate with levels of protein transitions during seed germination, which we also observed in the present study.

#### Protein Degradation

Protein degradation by proteolytic systems is an essential factor in many cellular processes, such as apoptosis, amino acid recycling and that avoids the detrimental effects (competition) associated with oxidized and misfolded protein accumulation. These proteins are degraded by the proteasome consisting of the 20S proteasome that degrades proteins in an ATP-independent manner and the ATP-dependent 26S proteasome that recognizes polyubiquinilated targeted proteins. This ATP-dependent reaction cascade involves the subsequent action of ubiquitin activating enzymes (E1s), ubiquitin conjugating enzymes (E2s), and ubiquitin protein ligase (E3s) enzymes (Vierstra, 2009; Marshall and Vierstra, 2019). In addition, the N-end rule pathway targets protein degradation and was shown to affect ABA sensitivity and promote seed germination (Holman et al., 2009; Zhang et al., 2018). In rice the RING Ub E3 ligase encoded by *OsCTR1* was shown to play an important role in drought tolerance; this study also showed that heterogeneous overexpression of *OsCTR1* in Arabidopsis entailed hypersensitive phenotypes with respect to ABA-responsive seed germination, seedling growth, and stomatal closure (Lim et al., 2014). Studies on endogenous plant proteases emerged and these enzymes are now admitted as taking part in the recognition and the subsequent degradation of targeted proteins (van der Hoorn, 2008). From our data, a cysteine endoproteinase was identified in the germinated rice embryo (Supplementary Table S4A). This protein is known to activate β-amylase through partial proteolysis and two cysteine endoproteinases, EP-A and EP-B, have been shown to digest hordein, the major barley seed storage protein (Guerin et al., 1992; Schmitt et al., 2013). Cysteine protease activities were related to the early remobilization of seed storage proteins during germination (Lu et al., 2015). In addition, we identified two isoforms of a proteasome subunit specifically at the germinated state (24 HAI) in rice embryos (Supplementary Table S4A). Likewise, RING-box (Really Interesting New Genes) protein 1a (RBX1A), is also germination specific (Supplementary Table S4A). These proteins are necessary for ubiquitin ligation activity of the multimeric cullin ring ubiquitin ligases. All RING finger proteins can bind to E2 ubiquitin-conjugating enzymes and possess E3 ubiquitin ligases, which promote targeted protein degradation. These enzymes are involved in many biological processes, such as DNA, RNA, and protein binding; nonetheless, germination specificity may pinpoint degradation machinery occurring during imbibition, necessary for proteome renewal from dry to imbibed seeds. Finally, LisH and RanBPM (Ran-binding protein in the microtubule-organizing center) domains containing protein (Complex GID, glucose-induced degradation) was also identified specifically at the germination stage (Supplementary Table S4A). This protein contains a LisH/CTHL (Lissencephaly type-1-like homology), CTHL being the (C-terminal domain of the LisH motif), and CRA domain. From previous studies in mammals and yeast, these domains demonstrated a proteosomal and/or lysosomal activity (Tomaru et al., 2010). CTLH identified in mammalian cells are homologous to glucose-induced degradation protein, which was previously identified as being involved in fructose biphosphatase degradation in both proteasome and vacuole in yeast (Regelmann et al., 2003; Tomaru et al., 2010). Studies on the Arabidopsis RanBPM containing LisH, CTHL, and CRA domains, disclosed a structural homology with CTHL complexes described in yeast and mammals and showed that the protein possesses a degradation activity (Tomaštíková et al., 2012). It will be important to specify the targets of these protein degradation-related enzymes for a better understanding of their roles in rice seed germination.

## Conclusion

In the present study, we captured the multi-omes (transcriptome, proteome, and metabolome) of embryo and endosperm tissues during rice germination and identified characteristic molecular responses in each tissue by integrative analysis. These approaches are highly complementary and provide spatial and temporal atlas of molecular and biochemical transitions during rice germination. The dynamic changes observed in the endosperm during the time course of germination suggest a very important role of this compartment to both protect the embryo and promote its growth potential. These data not only summarize existing processes important for germination but also provide evidence for germination-specific molecular responses and candidate core genes in the embryo, and the endosperm contribution to seed vigor. Future detailed functional analysis of the relevant genes and metabolites as well as the development of molecular markers specific for each tissue will be promising for the control of rice germination.

## Methods

### Biological Material

A total of 50 rice (*Oryza sativa* L. var. Nipponbare) seeds were used for germination assays at 30°C under dark and continuous oxygenation conditions in triplicate. Seeds with emerged coleoptiles were scored as germinated seeds at each time point. Water uptake values were calculated based on the dry and fresh weight of 10 bulks comprising 10 seeds. For multi-omics, three biological replicates of 150 whole seeds were used for each time point (0, 4, 8, 12, 16, 24 HAI). Rice seeds were dehulled and dissected with a sharp scalpel to separate isolated embryos (E0, E4, E8, E12, E16, E24) and isolated endosperms (A0, A4, A8, A12, A16, A24) during the time course of germination. The same samples were used for transcriptomic, proteomic, and metabolomic aiming to compare the multi-omics results as described below.

### Transcriptome Analysis

Total mRNAs were isolated from three replicates of 100 embryos and 50 endosperms and hybridizations on the Affymetrix GeneChip® Rice Genome Array (Affymetrix, Santa Clara, CA, USA) were performed as previously described (Galland et al., 2014, 2017). To obtain presence/absence calls for each probe, the CEL files were normalized by the MAS5 algorithm (Affymetrix). The CEL files were then normalized with the GC-RMA algorithm using the “gcrma” library available from the R Bioconductor suite of open-source software (Huber et al., 2015). Differentially expressed genes in the embryo and endosperm transcriptomes were detected by using a two-group *t*-test, followed by the Bonferroni method (adjusted *P*-value is < 0.01). All raw CEL files are available from the Gene Expression Omnibus under the accession GSE43780.

### Shotgun Proteomics

#### Protein Extraction and In-gel Digestion

For embryo protein extraction, three replicates of 50 embryos were ground in liquid nitrogen using a mortar and pestle. Total soluble proteins were extracted at room temperature in 400 μl thiourea/urea lysis buffer (7 M urea, 2 M thiourea, 6 mM Tris-HCl, 4.2 mM Trizma® base (Sigma-Aldrich, Lyon, France), 4% (w/v) CHAPS) supplemented with 50 μl of the protease inhibitor cocktail Complete Mini (Roche Diagnostics France, Meylan, France), 15 μl of dithiothreitol (DTT, 1 M, Sigma-Aldrich), 2 μl of DNase I (Roche Diagnostics), and 5 μl of RNase A (Sigma-Aldrich). For endosperm protein extraction, three replicates of 50 endosperms were ground in liquid nitrogen using a mortar and pestle and total soluble proteins were extracted at room temperature in 1 ml thiourea/urea lysis buffer (same composition as above) supplemented with 35 μl of DTT, 2 μl DNAse I, and 10 μl RNAse A. The protein extracts were left to agitate for 2 h at 4°C. All samples were then centrifuged at 20,000 g at 4°C for 15 min. The resulting supernatant was subjected to second clarifying centrifugation as above. The protein concentrations of the final supernatant were measured according to Bradford (1976) using Bovine Serum Albumin (BSA) as a standard. Twenty-five micrograms of embryo and endosperm soluble protein extracts (n = 3 biological replicates) were subjected to SDS-PAGE analysis with 10% acrylamide. Each lane was systematically cut into 16 slices and directly submitted to in-gel tryptic digestion with the Progest system (Genomic Solution) according to a standard trypsin protocol. Gel pieces were washed two times by successive separate baths of 10% acetic acid, 40% ethanol, and acetonitrile. They were then washed two times with successive baths of 25 mM NH4CO3 and ACN. Digestion was subsequently performed for 6 h at 37°C with 125 ng of modified trypsin (Promega) dissolved in 20% methanol and 20 mM NH4CO3. The peptides were extracted with 2% trifluoroacetic acid (TFA) and 50% ACN and then with ACN. Peptide extracts were dried in a vacuum centrifuge and suspended in 20 μl of 0.05% TFA, 0.05% HCOOH, and 2% ACN.

#### LC-MS/MS Analysis

Peptide separation by NanoLC was performed as described previously (Bonhomme et al., 2012). Eluted peptides were analyzed on-line with a Q-Exactive mass spectrometer (Thermo Electron) using a nano-electrospray interface. Peptide ions were analyzed using Xcalibur 2.1 with the following data-dependent acquisition parameters: a full MS scan covering 300–1,400 range of mass-to-charge ratio (m/z) with a resolution of 70,000 and an MS/MS step (normalized collision energy: 30%; resolution: 17,500). MS/MS Step was reiterated for the 8 major ions detected during the full MS scan. Dynamic exclusion was set to 45 s. For database searching, X!Tandem (Langella et al., 2017) was set up to search the 7th annotation of the Rice Genome Annotation Project database (Kawahara et al., 2013; Sakai et al., 2013) and a contaminant database (trypsin, keratins). Enzymatic cleavage was declared as a trypsin with two possible misscleavage. Cys carboxyamidomethylation was set to static modifications. Met oxydation was set as possible modifications. Precursor mass and fragment mass tolerance were 10 ppm and 0.02 Th, respectively. Only peptides with an E-value smaller than 0.1 were reported. Peptide quantification was performed by extracted ion current (XIC) using MassChroQ software (Valot et al., 2011). A 5 ppm precision windows was set for XIC extraction. The peptide ions not specific of a single protein were eliminated and reliably detectable ions that were detected at least twice out of the three biological replicates were used for the quantification. The Total Ionic Current (TIC) area under peak corresponding to the same ions was obtained for each peptide and then summed to get a protein abundance. The median normalization was used for the label-free data. The values of protein abundance were log2-transformed.

#### GO Enrichment Analysis for Differentially Accumulated Proteins

Differentially accumulated proteins during germination were determined by Student's *t*-test (*p* < 0.05) in embryos and endosperms, respectively. GO enrichment analysis for the up or down accumulated proteins was performed using the Functional Annotation tool in the CARMO (http://bioinfo.sibs.ac.cn/carmo/) with default parameters for thresholds (*p* < 0.05, fold enrichment > 2), followed by the Benjamini-Hochberg Procedure (adjusted *p* < 0.05). The values for -log10(adjusted *p*-values) and fold change of obtained GO terms in Biological Process were drawn using the ggplot2 package in R.

### Metabolome Analysis by Gas Chromatography Coupled to Mass Spectrometry (GC-MS)

Metabolite samples were obtained starting from three replicates of 100 rice seeds manually dissected in embryo and endosperm. Embryos and endosperms were ground with a mortar and pestle under liquid nitrogen and with a Cyclotec™ 1093 Sample Mill (FOSS, Hillerød, Danemark), respectively. All samples were lyophilized and around 20 mg dry weight (DW) of each sample were placed in 2 ml Safelock Eppendorf tubes (Eppendorf AG, Hamburg, Germany). All analysis steps, including extraction, derivatization, analysis, and data processing, were adapted from the original protocol described by Fiehn (2008) and following the procedure described by Avila-Ospina et al. (2017). The extraction solvent was prepared by mixing water:acetonitrile:isopropanol at the volume ratio 2:3:3 allowing to extract metabolites with a broad range of polarities. For derivatization step, N-methyl-N-trimethylsilyl-trifluoroacetamide (MSTFA; Sigma-Aldrich) was used in silylation procedure of metabolites. Samples were analyzed on an Agilent 7890A gas chromatograph coupled to an Agilent 5975C mass spectrometer. Raw Agilent datafiles were converted in NetCDF format and analyzed with AMDIS (Automated Mass Deconvolution and Identification System; http://chemdata.nist.gov/mass-spc/amdis/). A home retention indices/mass spectra library built from the NIST, Golm, and Fiehn databases and standard compounds was used for metabolites identification. Peak areas were then determined using the QuanLynx software (Waters, Milford, USA) after conversion of the NetCDF file in MassLynx format.

### Data Analysis Using R and MapMan

Figures were created using R version 3.6.3 (R Core Team, 2020; https://www.R-project.org/) using the version 1.3.0 of the tidyverse package suite (Wickham et al., 2019), the version 1.1 of the RColorBrewer (by Erich Neuwirth, 2014; https://CRAN.R-project.org/package=RColorBrewer), the version 1.0.0 of the patchwork library (by Thomas Lin Pedersen, 2019; https://cran.r-project.org/web/packages/patchwork/index.html). Supplementary Figure S3 was created using MapMap 3.6 (Schwacke et al., 2019) Code and data for Figures 1, 2, 4 can be found on GitHub https://github.com/mgalland/ (https://github.com/mgalland/rice-germination).

## Data Availability Statement

The datasets presented in this study can be found in online repositories. The names of the repository/repositories and accession number(s) can be found below: Transcriptomic data are available on the National Center for Biotechnology Information (NCBI) BioProject database under accession number GSE43780; Proteomic data are available on ProticDB, http://proteus.moulon.inra.fr/w2dpage/proticdb/angular/#/projects/163.

## Author Contributions

MG, IL, and LR designed and performed the experimental work. GCl completed the metabolome analyses. SB and SH performed the transcriptomic analysis while BV gathered the proteomic data. GCu and BC were involved in the preparation of samples. NS, MG, and LR were involved in data analysis and wrote the manuscript. All authors contributed to the article and approved the submitted version.

## Funding

This work was supported by the French Ministry of Industry (FUI, NUTRICE, agreement # 092906334). NS received funding from the European Union's Horizon 2020 research and innovation program under the Marie Skłodowska-Curie Grant Agreement No. [846387].

## Conflict of Interest

IL was employed by company MBCC Group. The remaining authors declare that the research was conducted in the absence of any commercial or financial relationships that could be construed as a potential conflict of interest.

## Publisher's Note

All claims expressed in this article are solely those of the authors and do not necessarily represent those of their affiliated organizations, or those of the publisher, the editors and the reviewers. Any product that may be evaluated in this article, or claim that may be made by its manufacturer, is not guaranteed or endorsed by the publisher.

## Supplementary Material

Supplementary Figure S1 Summary of main results obtained by transcriptomic, proteomic, and metabolomic approaches performed on rice dry embryo and endosperm during germination.

Supplementary Figure S2 Number of significant changes in metabolome during germination. **(A)** Embryo metabolites; **(B)** Endosperm metabolites. A one-way ANOVA was performed on each metabolite during the germination time course and significant (*p* < 0.05) metabolites were selected. The number of metabolites significantly up- (positive values) or down-accumulated (negative values) is shown for each pair of successive time points (*e.g.*, 0 HAI vs. 4 HAI, 4 HAI vs. 8 HAI, etc.) and for each tissue (embryo or endosperm).

Supplementary Figure S3 Seed-specific overview of differentially accumulated proteins. MapMan overview of differentially accumulated proteins between 0 and 24 HAI in the embryo or endosperm with a preferential abundance in the endosperm or embryo. The 261 embryo and 96 endosperm proteins with measurable and significant (*p* < 0.05) log_2_ ratios were displayed on a seed-specific visualization (Joosen et al., 2011). A total of 177 (embryo) and 71 (endosperm) proteins are visible. Red and blue colors represent proteins favorably accumulated at 24 HAI and 0 HAI. respectively. Only proteins present in MapMan rice MSU7 mapping file are displayed.

Supplementary Figure S4 Correlations between mRNA and protein accumulation profiles. Out of 1,659 and 597 embryo and endosperm genes for which a log2 fold change could be calculated between 24 and 0 HAI at both the transcript and protein level, the global Spearman correlation coefficient score was calculated. Both correlation coefficient and the number of genes are displayed for the embryo (left panel) and the endosperm (right panel).

Supplementary Figure S5 Phylogenetic tree of Arabidopsis and rice GID1 proteins. The tree was built based on amino acid sequence similarities from the 40 annotated rice GID1 and the three Arabidopsis GID1 proteins (framed square). The red circle indicates the rice *GID1* gene mostly studied in the literature. The blue triangle indicates the rice GID1 identified in germinated embryos in the present work. This figure was generated using COBALT: Multiple Alignment Tool (http://www.ncbi.nlm.nih.gov/tools/cobalt/cobalt.cgi?CMD=Web).

Supplementary Figure S6 Amino acid profiles in the embryo and endosperm during germination. The normalized median (*n* = 3) amino acid abundance was calculated for each time point and plotted on a relative (AU) scale for each tissue.

Supplementary Figure S7 The aspartate super pathway in rice embryo during germination. Transcript abundances are represented in squares for each locus. Colored squares indicate significant changes (*p* < 0.01, red: up accumulated, blue: down accumulated) for comparison between 2 times of imbibition: 0 vs. S5, 4 vs. 8, 8 vs. 12, 12 vs. 16, and 16 vs. 24 HAI. Up and down accumulated metabolites during germination are displayed, respectively, in red and blue boxes. The same color code is applied for proteins present in the total embryo proteome. AHB, acetohydroxybutyric acid, BCCAT: branched-chain amino acid aminotransferase, DHMV: 2,3-dihyroxy-3-methylvalerate.

Supplementary Table S1 Transcriptome of the germinating embryo and endosperm.

Supplementary Table S1A Embryo germination transcriptome data. According to the MAS5 algorithm, we detected 27,503 Affymetrix probes called “present” in at least one imbibition time and in at least two replicates (out of three) during embryo germination. Affymetrix control probes and locus non-specific probesets were removed from the analysis yielding a final number of 22,343 “present” probes corresponding to 18,389 unique rice loci. Then, the GCRMA normalized abundance of the embryo germination CEL files was retrieved for these 22,343 probes. Probe: identifier of the Affymetrix probeset, MSU_id: locus number according to the RGAP 7th annotation, Gene_model: representative transcript for the locus, MSU7_annotation: gene function according to the RGAP 7th annotation, Uniprot: protein description according to Uniprot (accessed 17 November 2012), P_EX: presence (P) or absence (A) of the probe in the three replicates after X hours of imbibition, Embryo_specific: “TRUE” indicates genes specifically detected in the embryo or “FALSE” if detected in both tissues (embryo and endosperm), E_X__*R*_Y_: GCRMA normalized probe intensity across the germination time course (X: hours after imbibition, Y: replicate number). The results of the differential analysis with log2 ratios (EYvsEX, Y > X) and associated Bonferroni-corrected *p*-value are indicated.

Supplementary Table S1B Endosperm germination transcriptome data. According to the MAS5 algorithm, we detected 24,695 Affymetrix probes called “present” in at least one imbibition time and in at least two replicates (out of three) during embryo germination. Affymetrix control probes and locus non-specific probesets were removed from the analysis yielding a final number of 22,052 “present” probes corresponding to 16,657 unique rice loci. Then, the GCRMA normalized abundance of the embryo germination CEL files was retrieved for these 20,052 probes. Probe: identifier of the Affymetrix probeset, MSU_id: locus number according to the RGAP 7th annotation, Gene_model: representative transcript for the locus, MSU7_annotation: gene function according to the RGAP 7th annotation, Uniprot: protein description according to Uniprot (accessed 17 November 2012), P_AX: presence (P) or absence (A) of the probe in the three replicates after X hours of imbibition, Endosperm_specific: “TRUE” indicates genes specifically detected in the embryo or “FALSE” if detected in both tissues (embryo and endosperm), AX_RY: GCRMA normalized probe intensity across the germination time course (X: hours after imbibition, Y: replicate number).

Supplementary Table S2 Differentially accumulated transcripts in the embryo and endosperm during rice seed germination.

Supplementary Table S2A A total of 7,665 up-accumulated transcripts in embryo during rice germination. The results of the differential analysis with log2 ratios (EYvsEX, Y > X) and associated Bonferroni-corrected *p*-value are indicated.

Supplementary Table S2B A total of 2,279 down-accumulated transcripts in embryo during rice germination. The results of the differential analysis with log2 ratios (EYvsEX, Y > X) and associated Bonferroni-corrected *p*-value are indicated.

Supplementary Table S2C A total of 5,926 up-accumulated transcripts in endosperm during rice germination. The results of the differential analysis with log2 ratios (AYvsAX, Y > X) and associated Bonferroni-corrected *p*-value are indicated.

Supplementary Table S2D A total of 2,103 down-accumulated transcripts in endosperm during rice germination. The results of the differential analysis with log2 ratios (AYvsAX, Y > X) and associated Bonferroni-corrected *p*-value are indicated.

Supplementary Table S3 Metabolome of embryo and endosperm during rice seed germination.

Supplementary Table S3A Single and averaged relative metabolite abundance data for the 121 detected metabolites in germinating embryos.

Supplementary Table S3B Comparison of relative metabolite abundance detected in rice embryo during germination time course.

Supplementary Table S3C Single and averaged relative metabolite abundance data for the 121 detected metabolites in endosperm during germination.

Supplementary Table S3D Comparison of relative metabolite abundance detected in rice endosperm during germination time course.

Supplementary Table S4 Shotgun proteomic data of the germinating embryo and endosperm.

Supplementary Table S4A The 2,315 proteins identified in the dry (E0) or 24 h-imbibed embryo (E24) from 25 μg of proteins. The peptides identified in the embryo were filtered to keep only the peptides specific to a single protein and a protein was considered as “present” if detected by at least one specific peptide in one biological replicate. These proteins correspond to 2,306 unique rice loci (Rice Genome Annotation Project version 7.0). The specificity of the protein is indicated (e.g., only present in the dry embryo).

Supplementary Table S4B Quantitative analysis of the 1,973 proteins common between the dry (E0) and 24h-imbibed embryo (E24) from 25 μg of proteins. The peptides identified in the embryo (ST1A) were filtered to keep only the peptides specific of a single protein and if the peptides were detected in at least 5 of the 6 samples (2 imbibition times x 3 replicates). MSU7_id: rice locus according to the RGAP 7.0 annotation, variance: result of the Fisher's test indicating if for each protein the variance is equal or different with *p* < 0.05, t_test: Student's *t*-test for each protein indicating significatively different mean with *p* < 0.05, log_2_ratio_E24vsE0: the log_2_ ratio between the protein mean abundance in E24 and E0 (a positive ratio significates that the protein is more abundant in 24 h-imbibed embryos as compared with dry embryos).

Supplementary Table S4C The 832 proteins identified in the dry (A0) or 24 h-imbibed endosperm (A24) from 25 μg of proteins. The peptides identified in the endosperm (ST3D) were filtered to keep only the peptides specific of a single protein and a protein was considered as “present” if detected by at least one specific peptide in one biological replicate. These proteins correspond to 829 unique rice loci (Rice Genome Annotation Project version 7.0). The specificity of the protein is indicated (e.g., only present in the dry endosperm).

Supplementary Table S4D Quantitative analysis of the 721 proteins common between the dry (A0) and 24h-imbibed endosperm (A24) from 25 μg of proteins. The peptides identified in the endosperm (ST1C) were filtered to keep only the peptides specific of a single protein and if the peptides were detected in at least 5 of the 6 samples (2 imbibition time x 3 replicates). MSU7_id: rice locus according to the RGAP 7.0 annotation, variance: result of the Fisher's test indicating if for each protein the variance is equal or different with *p* < 0.05, t_test: Student's *t*-test for each protein indicating significant different mean with *p* < 0.05, log_2_ratio_A24vsA0: the log_2_ ratio between the protein mean abundance in A24 and A0 (a positive ratio significates that the protein is more abundant in 24 h-imbibed endosperm as compared with dry endosperm).

Supplementary Table 5 Transcript correspondence with differentially accumulated proteins in the embryo and endosperm during rice germination.

Supplementary Table S5A The 265 significantly up-accumulated proteins in germinated rice embryos. Selection criteria: *p* < 0.05, log_2_ratio_E24vsE0 > 0. MSU7_id: rice locus according to the RGAP 7.0 annotation, Ontological class: obtained by a classification from agriGO analysis tool, t_test: Student's *t*-test for each protein indicating significant different mean with *p* < 0.05, log_2_ratio_E24vsE0: the log_2_ ratio between the protein mean abundance in E24 and E0.

Supplementary Table S5B The 229 transcripts corresponding to 229 up-accumulated proteins in rice embryo during germination. According to the MAS5 algorithm, we detected 27,503 Affymetrix probes called “present” in at least one imbibition time and in at least two replicates (out of three) during embryo germination. Affymetrix control probes and locus non-specific probesets were removed from the analysis yielding a final number of 22,343 “present” probes corresponding to 18,389 unique rice loci. Then, the GCRMA normalized abundance of the embryo germination CEL files was retrieved for these 22,343 probes. mRNA GCRMA abundance were normalized for each probe intensity by its maximum intensity over germination to get a value between 0 and 1. MSU_id: locus number according to the RGAP 7th annotation, E_X_: GCRMA normalized probe intensity across the germination time course with X standing for hours after imbibition (e.g., E_24_ stands for 24 HAI).

Supplementary Table S5C The 152 significantly down-accumulated proteins in germinated rice embryos. Selection criteria: *p* < 0.05, log_2_ratio_E24vsE0 < 0. MSU7_id: rice locus according to the RGAP 7.0 annotation, Ontological class: obtained by a classification from agriGO analysis tool, t_test: Student's *t*-test for each protein indicating significant different mean with *p* < 0.05, log_2_ratio_E24vsE0: the log_2_ ratio between the protein mean abundance in E24 and E0.

Supplementary Table S5D The 133 transcripts corresponding to 133 down-accumulated proteins in rice embryo during germination. According to the MAS5 algorithm, we detected 27,503 Affymetrix probes called “present” in at least one imbibition time and in at least two replicates (out of three) during embryo germination. Affymetrix control probes and locus non-specific probe sets were removed from the analysis yielding a final number of 22,343 “present” probes corresponding to 18,389 unique rice loci. Then, the GCRMA normalized abundance of the embryo germination CEL files was retrieved for these 22,343 probes. mRNA GCRMA abundance were normalized for each probe intensity by its maximum intensity over germination to get a value between 0 and 1. MSU_id: locus number according to the RGAP 7th annotation, E_X_: GCRMA normalized probe intensity across the germination time course with X standing for hours after imbibition (e.g., E_24_ stands for 24 HAI).

Supplementary Table S5E The 11 significantly up-accumulated proteins in germinated rice endosperm. Selection criteria: *p* < 0.05, log_2_ratio_A24vsA0 > 0. MSU7_id: rice locus according to the RGAP 7.0 annotation, Ontological class: obtained by a classification from agriGO analysis tool, t_test: Student's *t*-test for each protein indicating significant different mean with *p* < 0.05, log_2_ratio_A24vsA0: the log_2_ ratio between the protein mean abundance in A24 and A0.

Supplementary Table S5F The 11 transcripts corresponding to 11 up-accumulated proteins in rice endosperm during germination. According to the MAS5 algorithm, we detected 24,695 Affymetrix probes called “present” in at least one imbibition time and in at least two replicates (out of three) during endosperm germination. Affymetrix control probes and locus non-specific probe sets were removed from the analysis yielding a final number of 22,052 “present” probes corresponding to 16,657 unique rice loci. Then, the GCRMA normalized abundance of the endosperm germination CEL files was retrieved for these 20,052 probes. mRNA GCRMA abundance was normalized for each probe intensity by its maximum intensity over germination to get a value between 0 and 1. MSU_id: locus number according to the RGAP 7th annotation, A_X_: GCRMA normalized probe intensity across the germination time course with X standing for hours after imbibition (e.g., A_24_ stands for 24 HAI).

Supplementary Table S5G The 109 significantly down-accumulated proteins in germinated rice endosperm. Selection criteria: *p* < 0.05, log_2_ratio_A24vsA0 < 0. MSU7_id: rice locus according to the RGAP 7.0 annotation, Ontological class: obtained by a classification from agriGO analysis tool, t_test: Student's *t*-test for each protein indicating significant different mean with *p* < 0.05, log_2_ratio_A24vsA0: the log_2_ ratio between the protein mean abundance in A24 and A0.

Supplementary Table S5H The 96 transcripts corresponding to 96 down-accumulated proteins in rice endosperm during germination. According to the MAS5 algorithm, we detected 24,695 Affymetrix probes called “present” in at least one imbibition time and in at least two replicates (out of three) during endosperm germination. Affymetrix control probes and locus non-specific probesets were removed from the analysis yielding a final number of 22,052 “present” probes corresponding to 16,657 unique rice loci. Then, the GCRMA normalized abundance of the endosperm germination CEL files was retrieved for these 20,052 probes. mRNA GCRMA abundance were normalized for each probe intensity by its maximum intensity over germination to get a value between 0 and 1. MSU_id: locus number according to the RGAP 7th annotation, A_X_: GCRMA normalized probe intensity across the germination time course with X standing for hours after imbibition (e.g., A_24_ stands for 24 HAI).
