# Supplementary figures and images for: Multi-Omics Approaches Unravel Specific Features of Embryo and Endosperm in Rice Seed Germination
Source: Front Plant Sci. 2022 Jun 9;13:867263. doi: 10.3389/fpls.2022.867263 (PMC9225960; doi:10.3389/fpls.2022.867263)

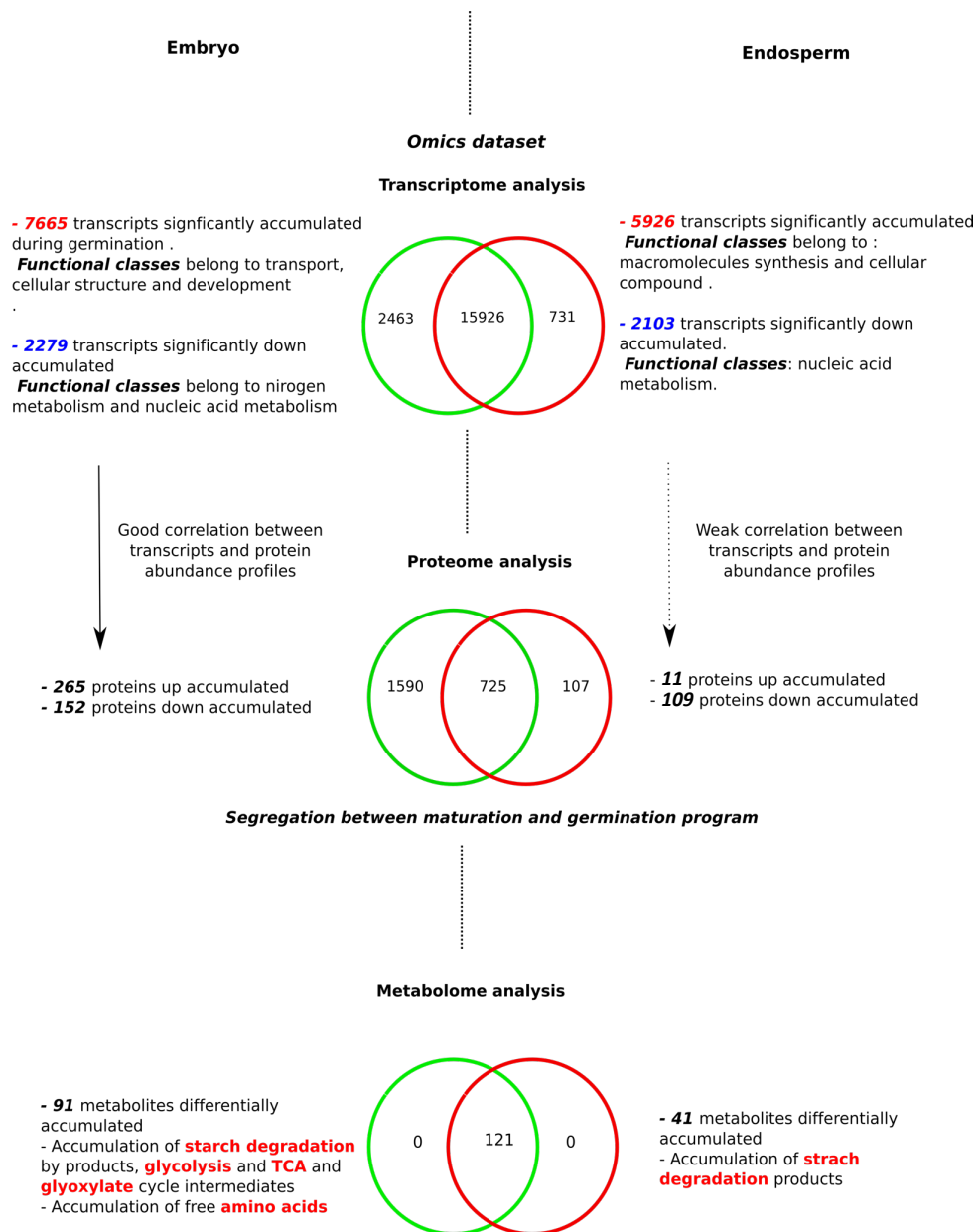

Figure S1

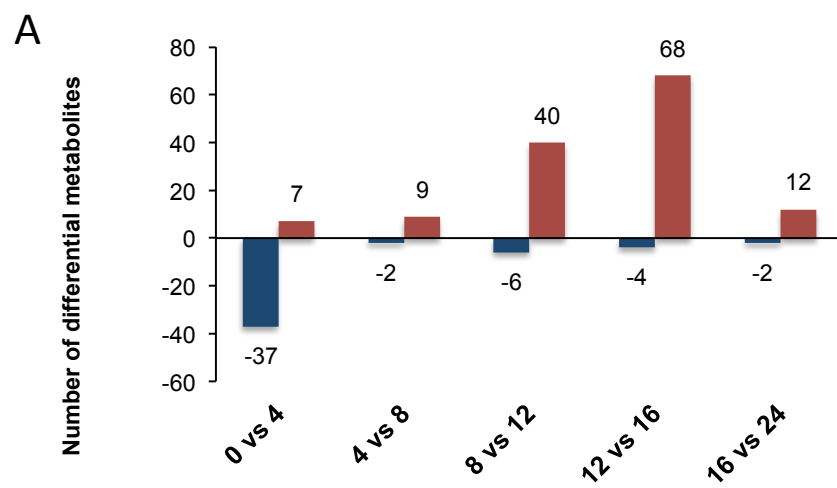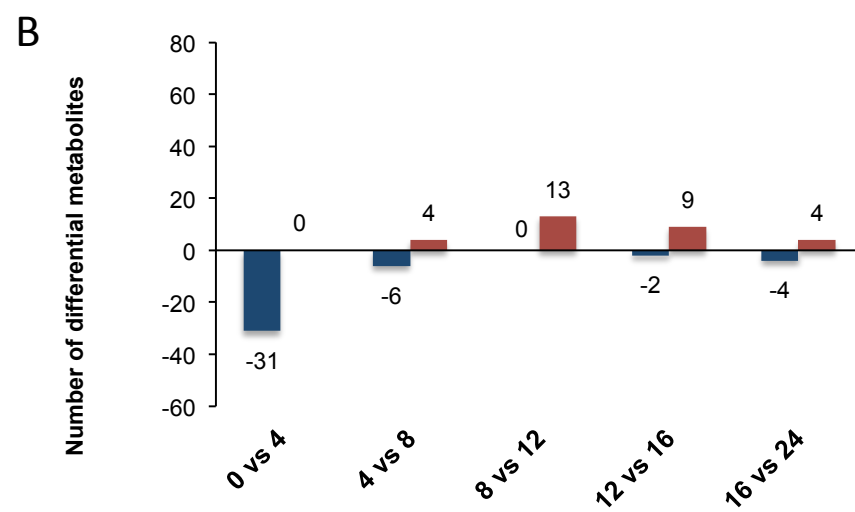

Figure S2

A

## Embryo

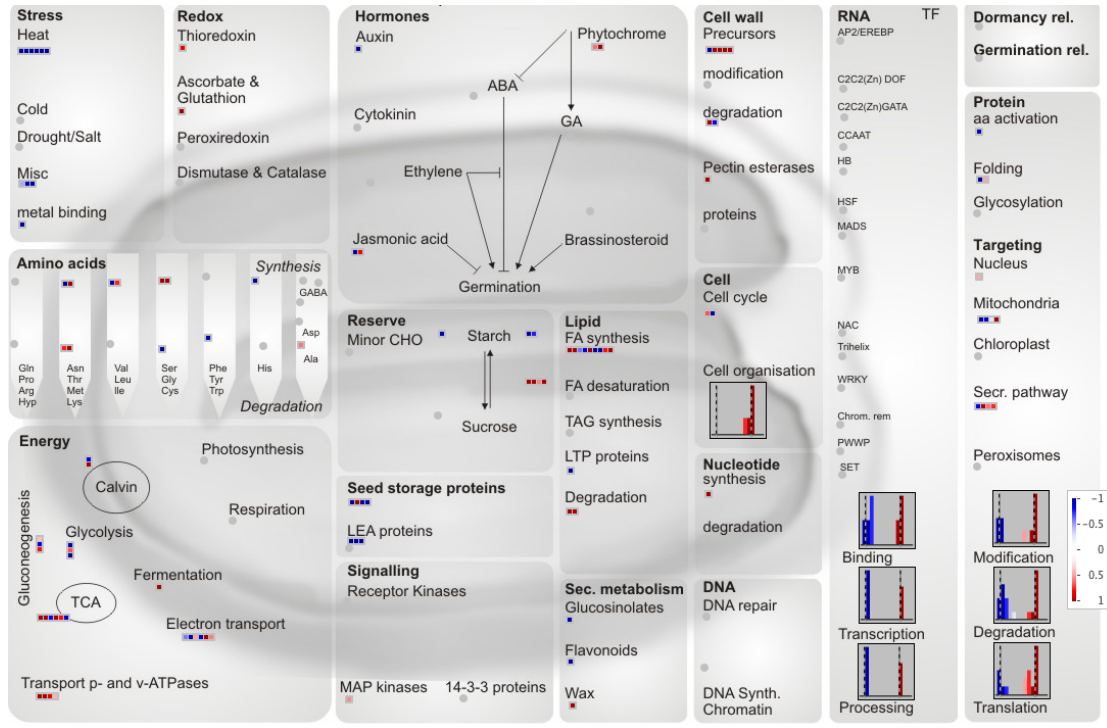

B

## Endosperm

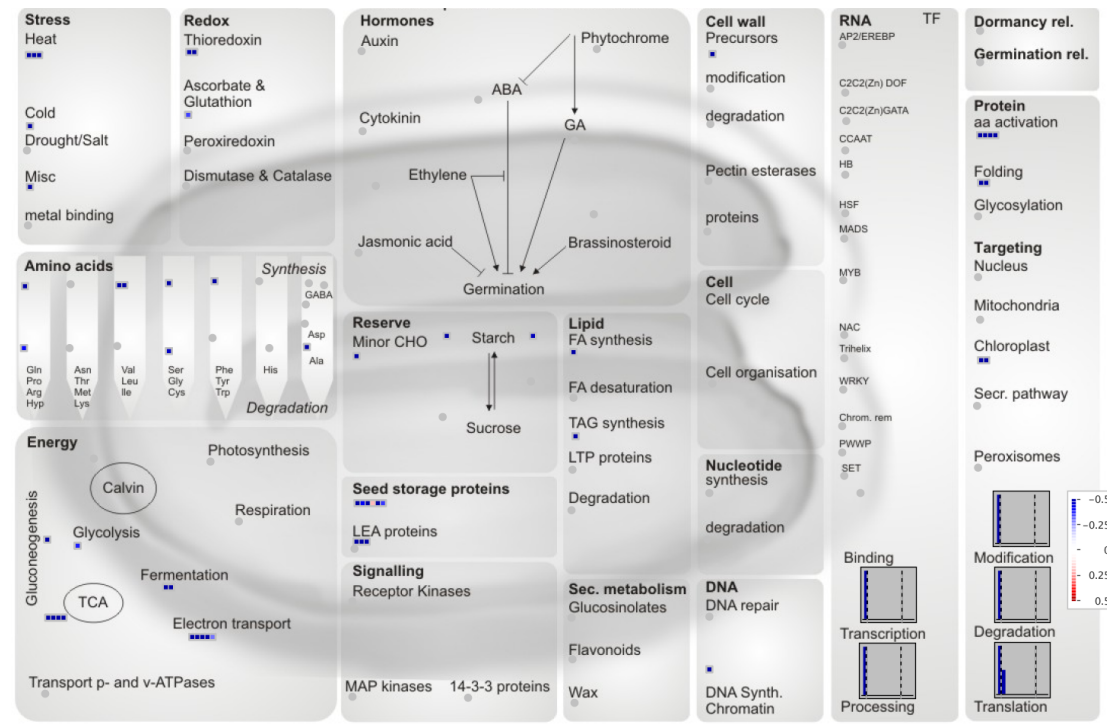

Figure S3

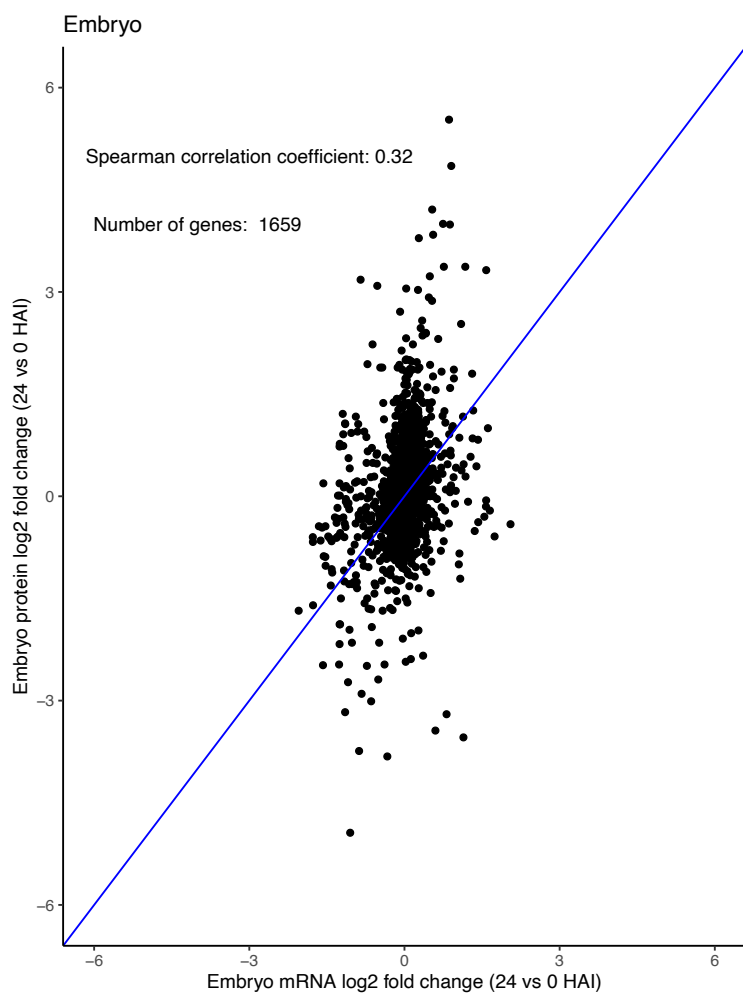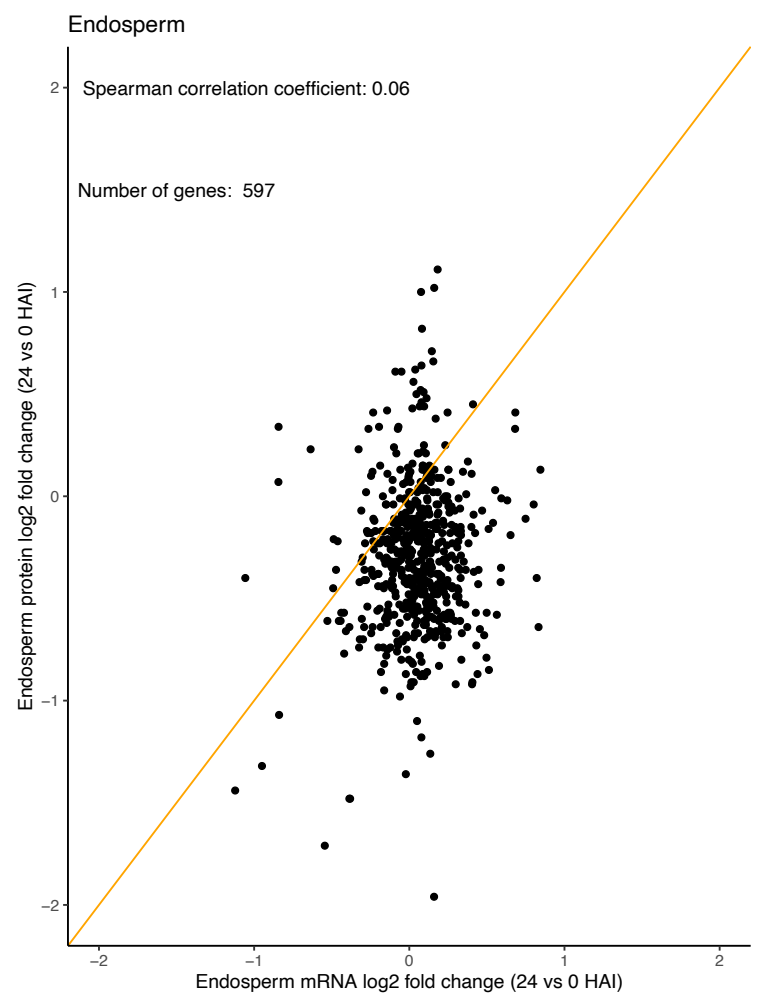

Figure S4

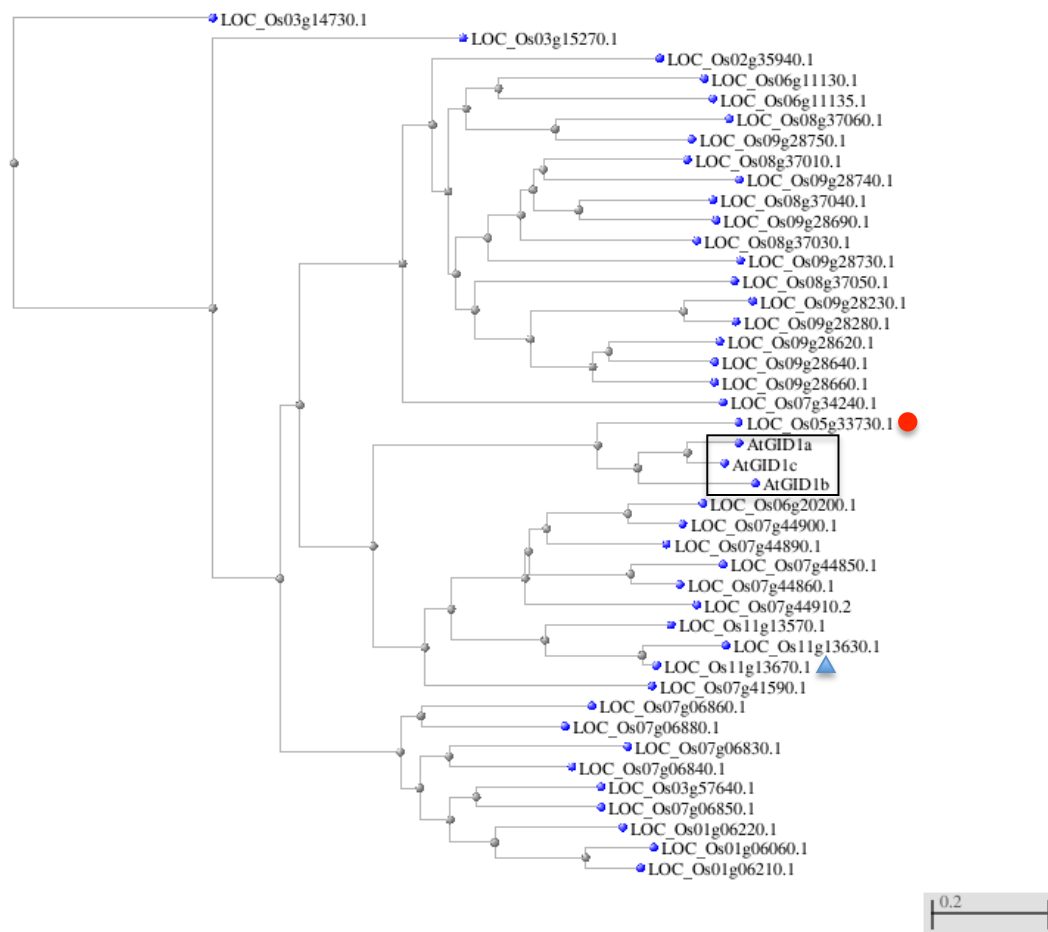

Figure S5

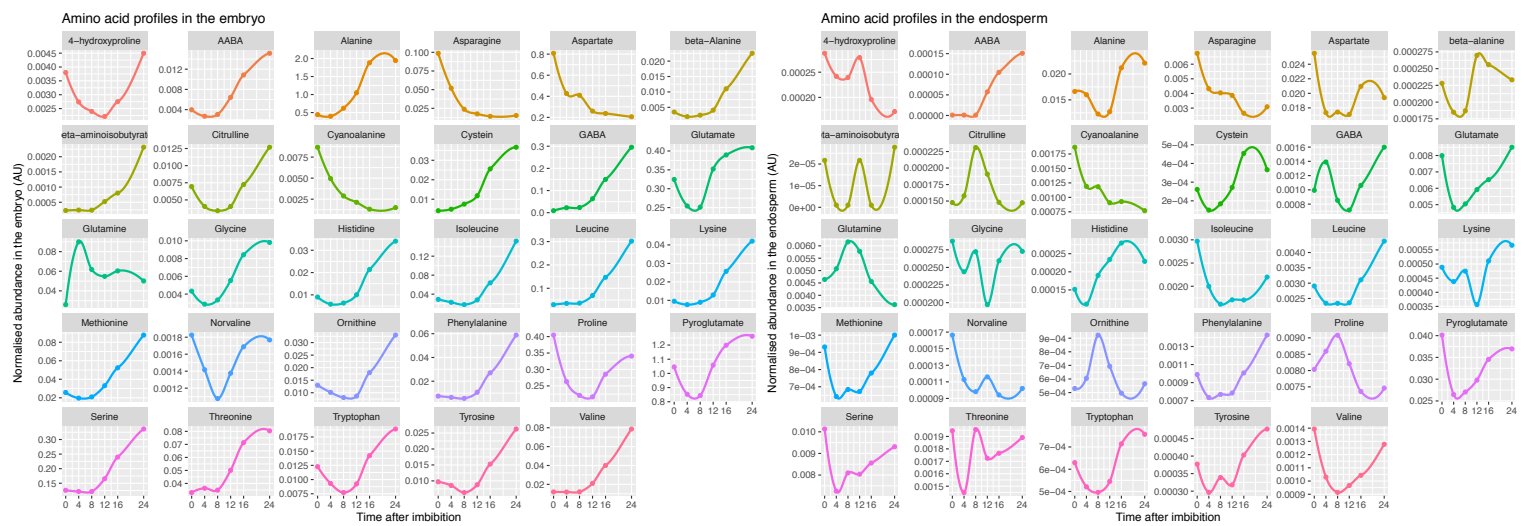

Figure S6

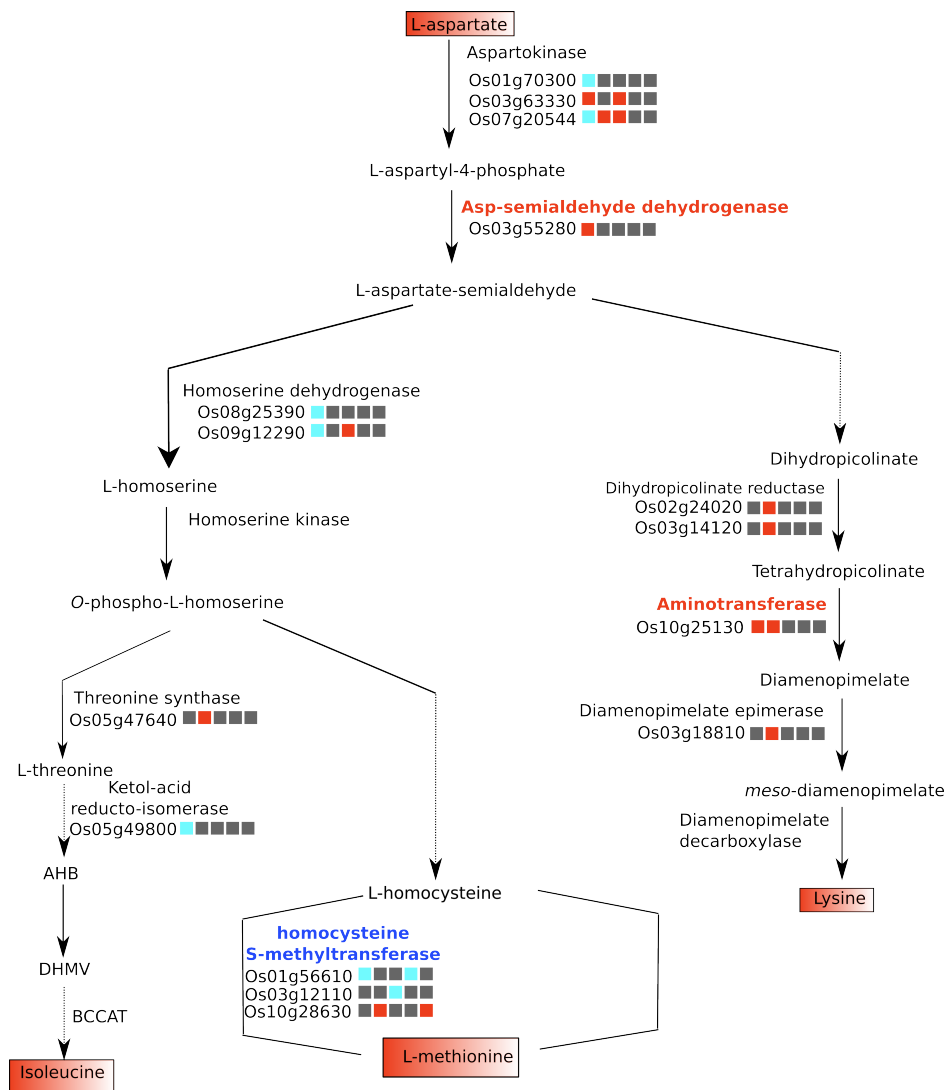

Figure S7

Supplement: Supplementary Figure S1 — Summary of main results obtained by transcriptomic, proteomic, and metabolomic approaches performed on rice dry embryo and endosperm during germination. [file Data_Sheet_1.PDF]
